# KDM4 Orchestrates Epigenomic Remodeling of Senescent Cells and Potentiates the Senescence‐Associated Secretory Phenotype

**DOI:** 10.1111/acel.70194

**Published:** 2025-08-24

**Authors:** Boyi Zhang, Qilai Long, Shanshan Wu, Qixia Xu, Shuling Song, Liu Han, Min Qian, Xiaohui Ren, Hanxin Liu, Jing Jiang, Jianming Guo, Xiaoling Zhang, Xing Chang, Qiang Fu, Eric W.‐F. Lam, Judith Campisi, James L. Kirkland, Yu Sun

**Affiliations:** ^1^ Shanghai Institute of Nutrition and Health University of Chinese Academy of Sciences, Chinese Academy of Sciences Shanghai China; ^2^ Department of Urology Zhongshan Hospital, Fudan University Shanghai China; ^3^ Institute of Health Sciences Shanghai Jiao Tong University School of Medicine & Shanghai Institutes for Biological Sciences, Chinese Academy of Sciences Shanghai China; ^4^ Department of Pharmacology Binzhou Medical University Yantai Shandong China; ^5^ Department of Orthopedic Surgery Xinhua Hospital, Shanghai Jiao Tong University School of Medicine Shanghai China; ^6^ Key Laboratory of Structural Biology of Zhejiang Province, School of Life Sciences Westlake University Hangzhou Zhejiang China; ^7^ Department of Surgery and Cancer Imperial College London London UK; ^8^ Buck Institute for Research on Aging Novato California USA; ^9^ Life Sciences Division Lawrence Berkeley National Laboratory Berkeley California USA; ^10^ Center for Advanced Gerotherapeutics Cedars‐Sinai Medical Center Los Angeles California USA; ^11^ Department of Medicine and VAPSHCS University of Washington Seattle Washington USA

**Keywords:** age‐related pathology, aging, cellular senescence, epigenetic modification, H3K9/H3K36 demethylation, KDM4

## Abstract

Cellular senescence restrains the expansion of neoplastic cells through several layers of regulation. We report that the histone H3‐specific demethylase KDM4 is expressed as human stromal cells undergo senescence. In clinical oncology, upregulated KDM4 and diminished H3K9/H3K36 methylation correlate with poorer survival of patients with prostate cancer after chemotherapy. Global chromatin accessibility mapping via assay for transposase‐accessible chromatin with high‐throughput sequencing, and expression profiling through RNA sequencing, reveals global changes of chromatin openness and spatiotemporal reprogramming of the transcriptomic landscape, which underlie the senescence‐associated secretory phenotype (SASP). Selective targeting of KDM4 dampens the SASP of senescent stromal cells, promotes cancer cell apoptosis in the treatment‐damaged tumor microenvironment, and prolongs survival of experimental animals. Our study supports dynamic changes of H3K9/H3K36 methylation during senescence, identifies an unusually permissive chromatin state, and unmasks KDM4 as a key SASP modulator. KDM4 targeting presents a new therapeutic avenue to manipulate cellular senescence and limit its contribution to age‐related pathologies, including cancer.

## Graphical abstract

The chromatin accessibility landscape of senescent cells is remodeled, with a set of activated transcription factors physically binding to the enhancers and promoters of senescence‐associated genes, including those encoding SASP factors. Events such as epigenomic reprogramming and KDM4‐mediated histone demethylation (H3K9/H3K36) enable SASP expression in senescent cells.

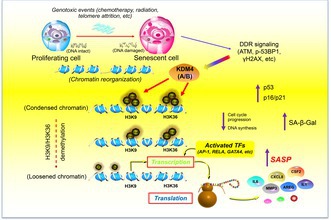

## 1 Introduction

Senescence is a self‐control mechanism that limits cell hyper‐proliferation and prevents neoplastic progression by implementing a stable, durable, albeit generally irreversible growth arrest. Senescent cells display profound alterations in nuclear and chromatin structure, expression patterns, and metabolic activities (Parry et al. 2018). Senescent cells also actively secrete a large array of proteins, many of which are pro‐inflammatory factors per se, a property collectively termed the SASP (Acosta et al. 2008; Coppe et al. 2008; Kuilman et al. 2008).

Several lines of evidence suggest that the SASP is essential for tissue repair, wound healing, embryonic development, and immune surveillance to clear senescent cells (Song et al. 2020). However, as a matter of evolutionary pleiotropy, this phenotype is mostly detrimental in numerous pathological settings (Sun et al. 2018). For example, in a local tumor microenvironment (TME), the secretome of lingering senescent cells can markedly promote malignancy of neighboring cancer cells, particularly chemoresistance, and cause chronic inflammation associated with many age‐related disorders (Furman et al. 2019; Gorgoulis et al. 2019; Khosla et al. 2020). Despite limited therapeutic efficacy‐promoting outcomes that depend on a given context (Ruscetti et al. 2020), the net effect of the SASP in advanced cancers far outweighs its beneficial contributions, ultimately accelerating disease progression (Gorgoulis et al. 2019; Song et al. 2020). Specifically, the harmful inflammation imposed by the SASP suggests that eliminating senescent cells or suppressing the SASP can be advantageous in multiple types of pathologies and not just cancer (Childs et al. 2017; Furman et al. 2019; Khosla et al. 2020).

The structure of chromatin is precisely regulated by epigenetic modifications such as DNA methylation and histone posttranslational modification (PTM), which functionally govern gene expression by changing chromatin organization and genome accessibility. The epigenetic landscape is prone to perpetual fluctuations throughout the lifespan and is profoundly altered in aging organisms (Gadecka and Bielak‐Zmijewska 2019). For instance, perturbations of histone methylation‐associated marks, such as loss of H3K9me3 and H3K27me3, are observed in normal human aging and premature aging diseases, such as Hutchinson‐Gilford progeria syndrome or Werner syndrome, suggesting that the global decline of heterochromatin may be a common feature of aging (Gruenbaum and Foisner 2015).

Lysine methylation is one of the major histone PTM forms that regulate chromatin structure. KDM4 (JMJD2) refers to a subfamily of demethylases that have a Jumonji domain and target histone H3 on lysine 9 and lysine 36 sites (H3K9 and H3K36), consisting of three 130‐KDa proteins (KDM4A‐C) and KDM4D, which is half the size and lacks double PHD and tandem Tudor domains as epigenome readers (Zhao et al. 2018). The role of the KDM4 subfamily is reported in multiple human cancer types, and small‐molecule inhibitors of KDM4 are being actively pursued to complement the existing arsenal of epigenetic drugs targeting DNA methyltransferases and histone deacetylases (Metzger et al. 2017). Despite accumulated data addressing the involvement of KDM4 in cancer progression, its functional implications and associated mechanisms in cellular senescence and critical activities such as the SASP development, specifically in non‐cancerous cells or normal tissues, remain underexplored. Understanding the significance of KDM4, a typical epigenetic factor that holds the potential to reprogram the chromatin landscape in senescent cells, is highly warranted in aging research and geriatric medicine.

## 2 Results

### 2.1 Histone H3 Lysine Sites in Senescent Cells Are Epigenetically Modified

**FIGURE 1:**
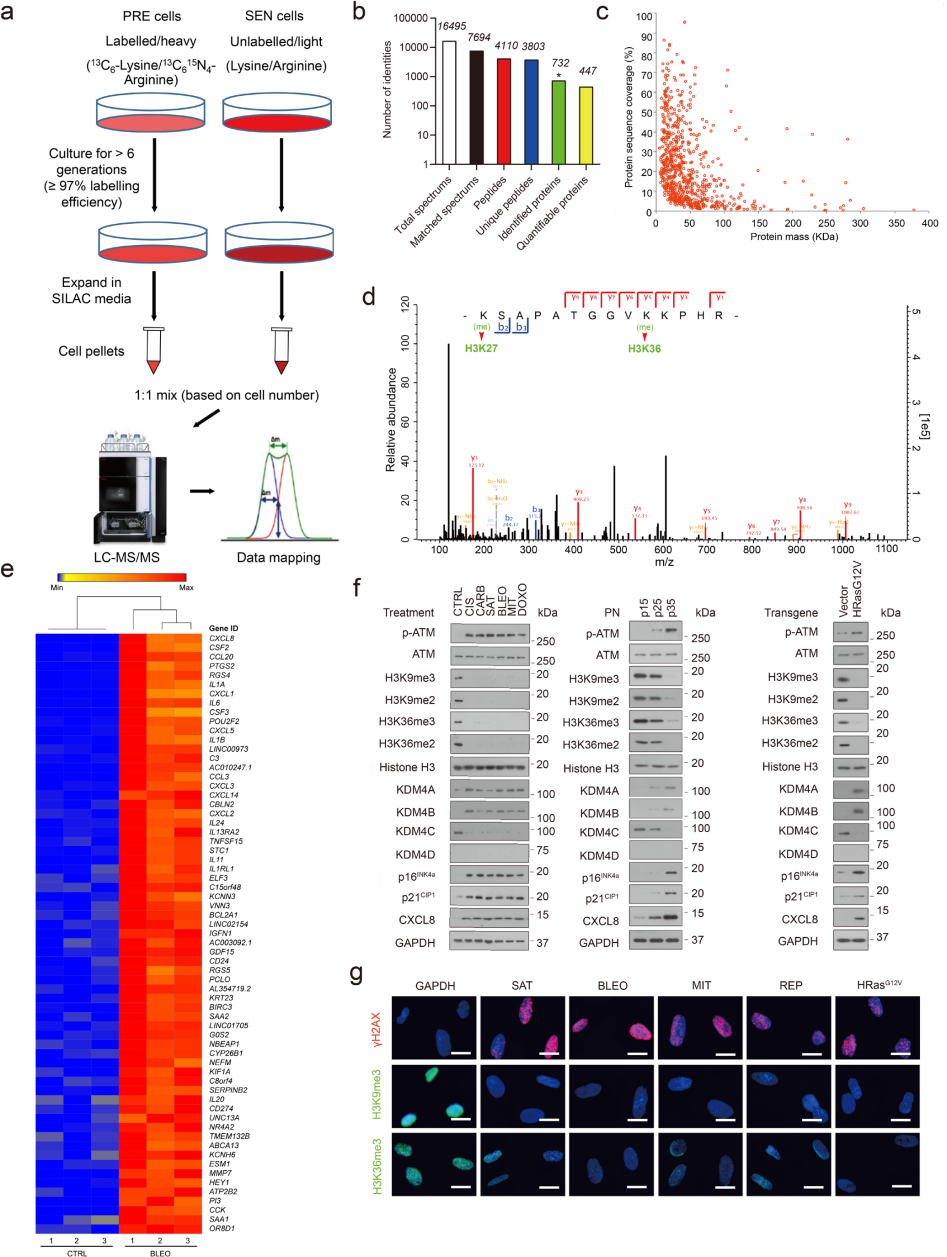
Histone H3 lysine sites are epigenetically modified upon cellular senescence. (a) Schematic of SILAC‐based identification of intracellular proteins in presenescent (PRE) versus senescent (SEN) cells of the human stromal line PSC27. (b) Column statistics of different categories of protein molecules in output data after SILAC analysis. An asterisk represents identified proteins (732). Data are representative of three independent experiments. (c) Scatterplot of proteins identified by the SILAC procedure. Protein sequence coverage was plotted against protein mass (447 quantifiable). (d) A representative plot derived from characterization of tandem mass spectrometry (MS/MS)‐based quantitative proteomics profiling. For MS scans, the *m/z* scan range was from 100 to 1100. Intact peptides were detected in the Orbitrap at a resolution of 70,000 with an automatic gain control target value of 1e5. (e) Heatmap depicting genes significantly upregulated in SEN cells after bleomycin (BLEO) treatment. CTRL, control. Genes are ordered by their expression fold change (highest on top) in PRE versus SEN cells after RNA‐seq. (f) Immunoblot analysis of key molecules in DDR, cellular senescence, and the SASP in PSC27 cells induced to senescence by chemotherapeutic agents (TIS), replicative exhaustion (RS) or oncogene‐induced senescence (OIS). PN, passage number. p15, p25, p35 represent different passages in culture. Vector denotes the empty control for human HRasG12V. H3K9me2/3 and H3K36me2/3 are histone H3 methylation markers. CIS, cisplatin; CARB, carboplatin; SAT, satraplatin; DOXO, doxorubicin. (g) IF staining of γH2AX, H3K9me3, and H3K36me3 in PSC27 cells after chemotherapeutic treatment (SAT, BLEO and MIT), replicative exhaustion (REP) or oncogene activation (HRasG12V). Scale bars, 5 μm. Data in (f, g) are representative of three independent biological replicates.

To establish an unbiased epigenetic profile of senescent cells at the protein level, we chose to use stable isotope labeling with amino acids (SILACs), a mass spectrometry (MS)‐based technique involving a nonradioactive isotope, to analyze senescent cells induced by bleomycin and their proliferating counterparts (control; Figure 1a). We examined independent biological replicates for each group and confirmed senescence in a primary normal human prostate stromal cell line (PSC27), which consists predominantly of fibroblasts (Sun et al. 2012). Overall, we detected 732 intracellular proteins with 95% confidence, of which 447 entities are quantifiable (Figure 1b,c) (Tables S1 and S2).

Among the differentially expressed proteins we identified, 87 showed a significant increase and 29 displayed a significant decrease in abundance in senescent cells (> twofold; *p* < 0.05), respectively (Table S1). However, when analyzing PTM sites differentially modified between presenescent and senescent cells, we noticed a reduction of histone H3.2 signals in the categories of both dimethylated and trimethylated proteins, namely H3K27 and H3K36 (Figure 1d, Tables S2 and S3). As histone PTMs can alter the architecture of chromatin and are implicated in the epigenetic regulation of senescent cell‐associated phenotypes (Cheung et al. 2018), we interrogated the possibility of systematic or general changes at histone H3 sites, and whether they are causal for specific consequences during senescence.

To address these questions, we primarily focused on the major forms of PTMs occurring at histone H3.2, including methylation. As it is known that the level of H3K27me3, a repressive histone mark mainly enriched in heterochromatin, tends to diminish in senescent human fibroblasts via an autophagy/lysosomal pathway (Dou et al. 2015; Ivanov et al. 2013), here we paid special attention to H3K36, whose demethylation and resulting implications are generally less reported for senescence (Tanaka et al. 2020). To substantiate the data from proteomics assays, we performed whole transcriptomic assessment with RNA sequencing (RNA‐seq). The data showed a typical SASP expression profile, as evidenced by upregulation of multiple pro‐inflammatory factors and activation of senescence‐associated signaling pathways (Figure 1e and Figure S1a), confirming entry into cellular senescence after genotoxic stress. Expression assessment of classic histone‐specific methylases and demethylases revealed a distinct upregulation pattern of epigenetic factors, specifically the KDM4 family (including members A, B, C, and D), although the significance of only A and B was confirmed by quantitative PCR with reverse transcription (RT‐qPCR; Figure S1b,c). To build on these findings, we used several chemotherapeutic drugs including cisplatin, carboplatin, satraplatin, mitoxantrone (MIT) and doxorubicin, in addition to bleomycin to treat PSC27 cells (Figure 1f). Additionally, we exposed cells to replicative senescence and oncogene‐induced senescence (HRas^G12V^; Figure 1f). We observed prominent cellular senescence and remarkable induction of KDM4 family members A and B (Figure 1f, Figure S1d,e). Immunoblot analysis indicated phosphorylation of ATM, enhanced expression of p16^INK4a^, p21^CIP1^, and CXCL8 (interleukin (IL)‐8, a hallmark SASP factor), but a diminished methylation pattern (including trimethylation and dimethylation) at the H3K36 site (Figure 1f). As KDM4 selectively demethylates both H3K9 and H3K36, an activity implicated in key cellular processes including DNA damage response (DDR), cell cycle regulation, and senescence (Ipenberg et al. 2013), we examined H3K9 methylation in parallel. Not surprisingly, H3K9 exhibited a demethylation tendency largely resembling that of H3K36 throughout all cell‐based assays (Figure 1f).

Upon immunofluorescence (IF) staining, we observed markedly reduced signals of both H3K9me3 and H3K36me3, sharply contrasting with γH2AX, a canonical marker of DDR foci at DNA double‐strand breaks (DSBs), the expression of which was apparently enhanced in DNA‐damaged PSC27 cells (Figure 1g). The data suggested that the loss of epigenetic PTM marks at histone H3.2, specifically those imaging H3K9 and H3K36 methylation status, is accompanied by increased KDM4 expression (mainly A and B isoforms, hereafter KDM4A/B), and occurs consistently in senescent stromal cells as a potentially general tendency, though caused by an unknown mechanism and with unclear biological consequences.

We next investigated these senescence‐associated epigenetic changes in HFL1, a primary human lung fibroblast line, and found H3K9/H3K36 demethylation was largely reproduced (Figure S2a–c). To further expand, we examined two sets of typical human cancer cell lines including those derived from prostate and lung malignancies, respectively. However, in contrast to data derived from stromal cells, epigenetic alterations including histone H3.2 demethylation and KDM4 expression appeared to vary substantially among cancer cell lines, despite consistently induced cellular senescence after drug treatment (Figure S2d,e). In addition, we noticed that ectopic expression of p16^INK4a^ in PSC27 or HFL1 cells can partially recapitulate epigenetic changes as observed in genotoxic settings, although signals were mostly marginal (Figure S2f,g). In contrast to the case of oncogenic activation (HRas^G12V^), p16 did not induce SASP expression (Figure S2h,i), which was basically in line with previous findings (Coppe et al. 2011). These data suggest that stromal cells, rather than their malignant counterparts, may commonly share an epigenetic program inherently correlated with cellular senescence, while both epigenetic remodeling and DDR signaling seem to be essential for the development of a mature pro‐inflammatory phenotype of senescent cells, a feature that can alter the microenvironment and has wide implications in organismal aging and associated pathologies.

### 2.2 Stromal Expression of KDM4A/B in Damaged Tumor Microenvironment Correlates With Adverse Clinical Outcomes

**FIGURE 2:**
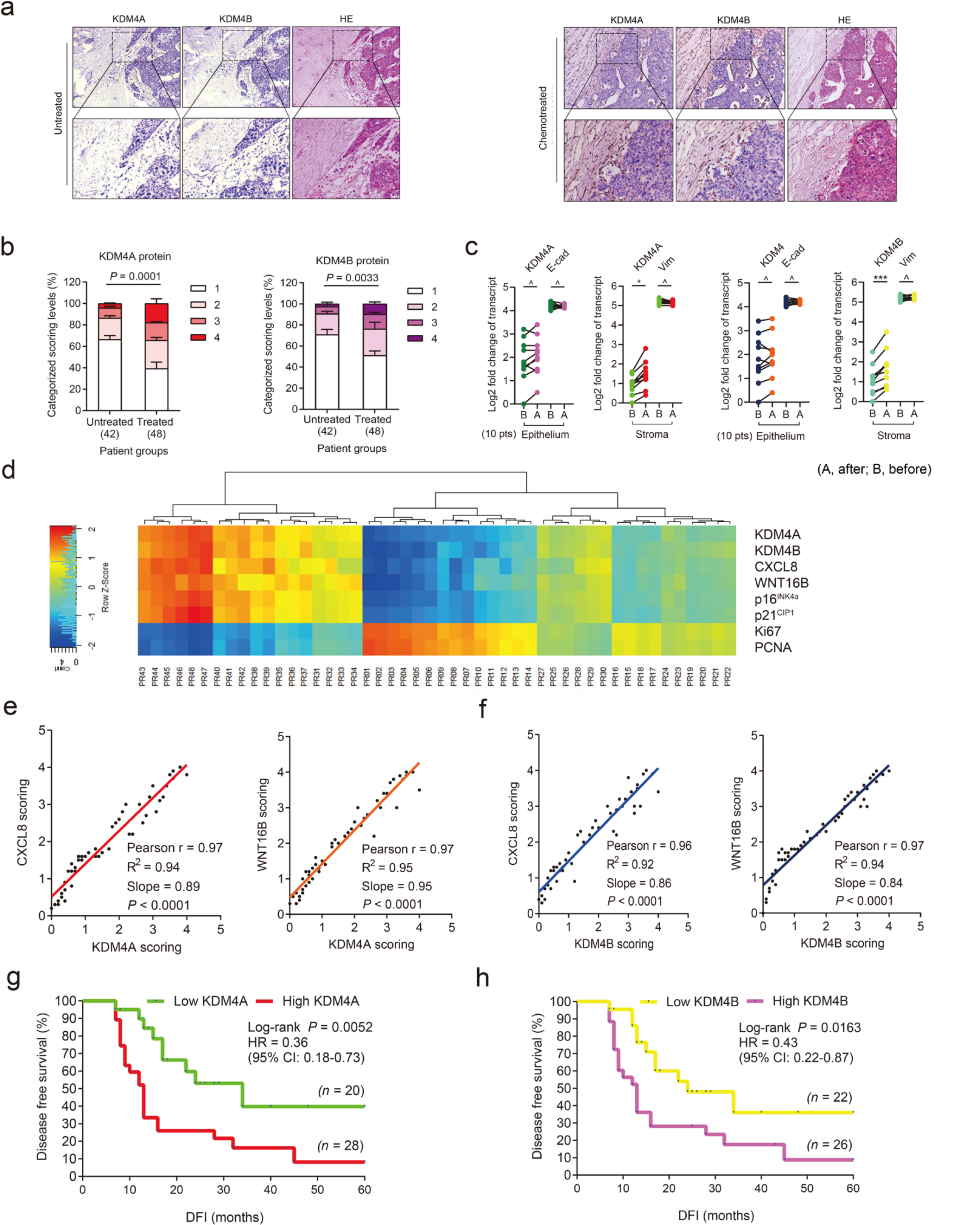
KDM4A and KDM4B are expressed in human prostate tumor stroma and correlate with adverse clinical survival. (a) Histological images of KDM4A/B in human PCa tissues. Left, before chemotherapy. Right, after chemotherapy. Scale bars, 100 μm. HE, hematoxylin and eosin. (b) Pathological assessment of stromal KDM4A/B in PCa tissues (42 untreated versus 48 treated). Participants are pathologically assigned into four categories per IHC staining intensity in stroma: 1, negative; 2, weak; 3, moderate; 4, strong expression. *p* values were determined by two‐way analysis of variance (ANOVA) with Bonferroni's post hoc test. (c) Comparative analysis of KDM4A/B before (B) and after (A) chemotherapy. Each dot represents an individual, with (B and A) data points connected to allow direct profiling per individual (*n* = 10 participants). *p* values were determined by two‐sided unpaired *t*‐test. ^#^ *p* > 0.05. *p < 0.05; from left to right, *p* = 0.6152, *p* = 0.8451, *p* = 0.0486, *p* = 0.5253, *p* = 0.7660, *p* = 0.8279, *p* = 0.0179, and *p* = 0.4612. (d) Landscape of pathological correlation between KDM4A/B, CXCL8, WNT16B, p16^INK4a^, p21^CIP1^, Ki67, and PCNA in stroma of individuals with PCa after chemotherapy. Scores derived from histological assessment per factor (48 patients after treatment). (e) Statistical correlation (Pearson analysis) between pathological scores of KDM4A and CXCL8/WNT16B expressed in 48 individuals with PCa. *p* values were determined by two‐sided unpaired *t*‐test and adjusted for multiple comparisons. (f) Similar to data in (e) but showing the correlation of KDM4B and CXCL8/WNT16B. (g) Kaplan–Meier analysis of individuals with PCa. DFS stratified according to KDM4A expression (low, average score < 2, green line, *n* = 20; high, average score ≥ 2, red line, *n* = 28). (h) Kaplan–Meier analysis of individuals with PCa. DFS stratified according to KDM4B expression (low, average score < 2, yellow line, *n* = 22; high, average score ≥ 2, pink line, *n* = 26). Data in (a) are representative of three independent biological replicates. Statistics of (g and h) survival curves derived from Kaplan–Meier analysis, with *p* values calculated using a two‐sided log‐rank (Mantel‐Cox) test. Data in b are shown as mean ± SD. and representative of three biological replicates. HR, hazard ratio; CI, confidence interval; DFI, disease‐free interval.

Given the inducible expression of KDM4A/B in senescent cells under in vitro conditions, we asked whether the results are reproducible in clinical settings. We first investigated the biospecimens derived from a cohort of individuals with prostate cancer (PCa) and observed remarkable upregulation of KDM4A/B in prostate tumor tissues after chemotherapy, specifically in contrast to the samples collected before treatment (Figure 2a). Interestingly, upregulated KDM4A/B were generally localized in the stromal compartments rather than their adjacent cancer epithelium, which appeared to have limited or no signals.

Expression of KDM4A/B in prostate tissues before and after chemotherapy was quantitatively substantiated by a pre‐established pathological evaluation protocol for precise assessment of a target protein expression according to its immunohistochemistry (IHC) staining intensity (Figure 2b). We further analyzed a subset of participants whose pre‐chemotherapy and post‐chemotherapy samples were both accessible, by performing laser capture microdissection (LCM)‐based transcript assays per cell lineage. The data showed significant upregulation of KDM4A/B in the stroma, but not their nearby cancer epithelium in posttreatment biospecimens (*p* < 0.05 vs. *p* > 0.05; Figure 2c). We further noticed the expression dynamics of KDM4A/B in damaged TME essentially parallel those of CXCL8 and WNT16B, two hallmark SASP factors (Figure 2d). This tendency was phenocopied straightly by p16^INK4a^ and p21^CIP1^, typical markers of senescence, but reversely by Ki67 and PCNA, indicators of cell proliferation (Figure 2d). Correlation between KDM4A/B and CXCL8/WNT16B expression in the damaged TME was further supported by pathological evaluation of their expression scores in participants after treatment (Figure 2e,f). More importantly, Kaplan–Meier analysis of participants with PCa who were stratified according to KDM4A/B expression in tumor stroma suggested a significant, albeit negative, correlation between KDM4A/B and disease‐free survival (DFS) in the treated cohort (Figure 2g,h).

We also analyzed the situations of KDM4C/D, the other members of the KDM4 subfamily. Pathological data suggested that neither molecule was upregulated in tumor stroma after chemotherapy, while expression profiling *per* cell lineage showed no changes in stromal or epithelial compartments (Figure S3a–d). Furthermore, pathological stratification according to KDM4C/D expression in tumor stroma failed to reveal potential correlations between KDM4C/D and DFS in the treated cohort (Figure S3e,f).

Given the prominent induction of KDM4A/B in tumor stroma of individuals with cancer after treatment, we questioned if there were possible changes in methylation levels of H3K9 and H3K36, two major targets of demethylases KDM4A/B in human cells. IHC assessment of participant samples suggested attenuated H3K9me3 and H3K36me3 signals in stromal cells, whereas neighboring cancer cells in the disease foci appeared largely unaffected (Figure S4a–d). LCM‐supported comparative expression assays *per* cell lineage confirmed the declining pattern of H3K9me3 and H3K36me3 in stromal cells, but not their epithelial counterparts in the TME after treatment (Figure S5a). Of note, there was a largely reverse correlation between KDM4A/B expression and H3K9/H3K36 trimethylation, as indicated by pathological evaluation of target signals in posttreatment samples (Figure S5b). Pearson statistical analysis further substantiated these inherent links (Figure S5c). As a special point, we noticed a lower level of H3K9/H3K36 trimethylation in stromal compartments correlated with a shorter survival of individuals with PCa in the post‐chemotherapy stage (Figure S5d,e), implying a potential mechanism associated with these epigenetic marks and driving adverse consequences, specifically cancer mortality.

### 2.3 KDM4A/B Expression Is Accompanied by Decreased H3K9/H3K36 Methylation in Senescent Cells

**FIGURE 3:**
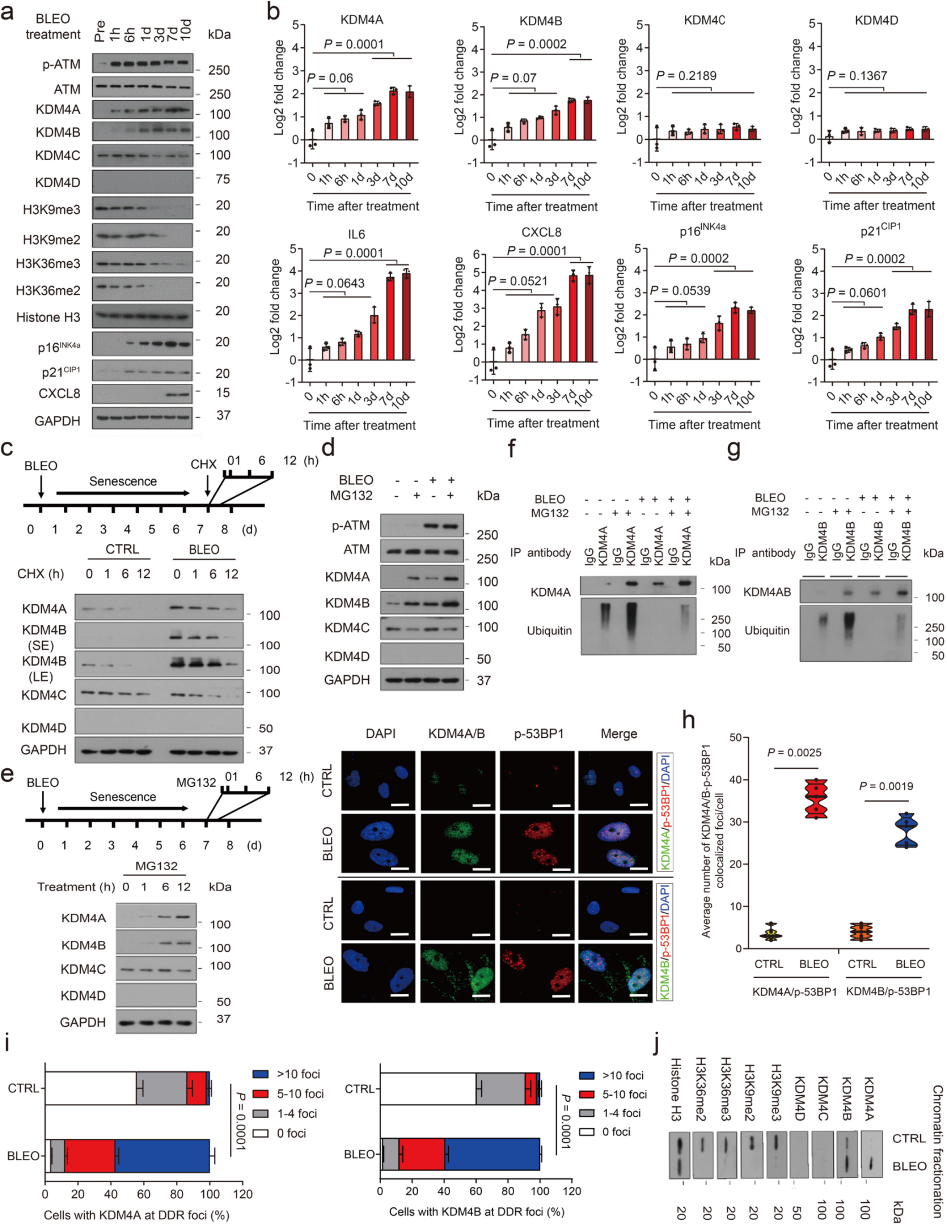
KDM4 expression is regulated at the posttranslational in senescent cells. (a) Time‐course analysis of KDM4 expression in PSC27 cells after BLEO treatment. Cell lysates were collected at indicated time points after treatment and subject to immunoblot assays. GAPDH, loading control. (b) Time‐course measurement of transcript expression of KDM4 subfamily members and IL‐6, CXCL8, p16^INK4a^, and p21^CIP1^ in stromal cells after treatment. *p* values were determined by one‐way ANOVA and adjusted for multiple comparisons. (c) Immunoblot of KDM4 expression in stromal cells treated by BLEO and/or CHX. The upper panel shows a schematic representation of experimental design and timeline. Cell lysates were collected at the indicated time points after CHX addition to media. (d) Immunoblot of KDM4 expression in stromal cells treated by BLEO and/or MG132. Cell lysates were collected after control or BLEO‐induced senescent cells were treated by MG132 for 12 h. (e) Immunoblot appraisal of KDM4 protein levels in PSC27 cells treated by MG132. Cell lysates were collected at time points as indicated by the experimental scheme. (f) Evaluation of KDM4 protein PTM via IP followed by immunoblot assays. Anti‐KDM4A/B was used for IP, with precipitates subjected to immunoblot assay with KDM4A/B antibody. Anti‐ubiquitin was used to probe the ubiquitination profile of KDM4A/B. (g) IF staining of KDM4A/B and p‐53BP1 in stromal cells. Cells were treated by BLEO and subjected to IF staining 7 days later. KDM4A/B, green; p‐53BP1, red. Nuclei (DAPI), blue. Scale bars, 5 μm. (h) Comparative statistics of the average number of foci where KDM4A/B and p‐53BP1 are colocalized in damaged PSC27 per cell. (i) Statistics of stromal cells displaying nuclear colocalization of KDM4A/B and p‐53BP1 in control versus SEN cells. *p* values were determined by two‐way ANOVA with Bonferroni's post hoc test. (j) Immunoblot assessment of KDM4 and H3K9/H3K36 methylation after chromatin fragmentation. Histone H3, loading control for nuclear lysates. Data in all bar plots are the mean ± SD and representative of three biological replicates. Data in (a, c–e, f, g and j) are representative of three replicates. In (b and h), *p* values were determined by two‐sided unpaired *t*‐test and adjusted for multiple comparisons.

We next performed a time‐course expression assay with stromal cell lysates collected at individual time points after genotoxicity‐induced senescence. In contrast to DDR signaling, which appears to be an acute response, KDM4A/B protein levels gradually increased within the first 7 days after treatment before entering a plateau. KDM4C was slightly enhanced upon DNA damage but seemingly declined after the first 6 h until approaching a stable phase, whereas KDM4D was essentially undetectable at the protein level throughout the assays (Figure 3a). Transcription analysis of KDM4 largely reproduced the data from immunoblots, with KDM4A/B levels progressively increasing in senescent cells, while KDM4C/D did not (Figure 3b). Of note, the expression pattern of KDM4A/B was indeed similar to that of SASP hallmark factors (IL6 and CXCL8) and cyclin‐dependent kinase inhibitors (p16^INK4a^ and p21^CIP1^; Figure 3b).

To dissect the mechanism supporting KDM4 protein expression, we treated cells with cyclohexamide (CHX), a pan‐protein synthesis inhibitor. In contrast to control cells, BLEO‐induced senescent cells exhibited increased KDM4A/B expression, though signals in each case diminished in a time course upon CHX treatment, suggesting KDM4A/B are subject to protein turnover in these cells (Figure 3c). In the presence of MG132, a cell‐permeable proteasome inhibitor, protein levels of KDM4A/B were elevated, thus confirming that both factors are indeed vulnerable to proteasome‐mediated degradation (Figure 3d). Distinct from the case of KDM4C/D, which remained largely unchanged, MG132 markedly increased KDM4A/B levels, thus further supporting their proteasome‐regulated nature (Figure 3e). Subsequently, immunoprecipitation (IP) from total cell lysates indicated enhanced KDM4A/B upon MG132 treatment, but with decreased intensities of ubiquitin‐mediated PTM upon BLEO treatment, suggesting that increased KDM4A/B proteins are partially correlated with a ubiquitin/proteasome‐escaping mechanism in senescent cells after genotoxic stimuli (Figure 3f). Such a pattern sharply contrasts with that of TRAF6, a molecule exhibiting ubiquitin E3 ligase activity for diverse substrates including itself (auto‐ubiquitination) upon cellular stress and senescence (Ji et al. 2016; Meng et al. 2019; Zhang et al. 2018), and makes the case of KDM4A/B an interesting topic that warrants future investigation. Together, these data suggest operation of an intracellular mechanism specifically protecting KDM4A/B against ubiquitination‐mediated protein degradation in senescent cells.

Upon IF‐based cell staining, we noticed enhanced levels of KDM4A/B in the nuclei of PSC27 (Figure 3g). Interestingly, both factors are substantially co‐localized with p‐53BP1, one of the canonical markers of DDR foci, signals of which displayed a significant increase in DNA‐damaged cells (Figure 3g,h). Notably, the percentage of cells detected with KDM4A/B at DDR foci was considerably enhanced after BLEO treatment (Figure 3i). To establish the subcellular localization of KDM4A/B upon cellular senescence, we examined cell lysates with chromatin fractionation. Immunoblot analysis indicated that both factors were significantly elevated in the chromatin fractions of senescent cells relative to their proliferating counterparts (Figure 3j). Thus, KDM4A/B are regulated by mechanisms involving both PTM‐associated and ubiquitin/proteasome‐eluding pathways, with a potential but yet unknown epigenetic role in the nuclear compartment of senescent cells.

### 2.4 KDM4A/B Functionally Regulate the Senescence‐Associated Secretory Phenotype Without Affecting Senescence

**FIGURE 4:**
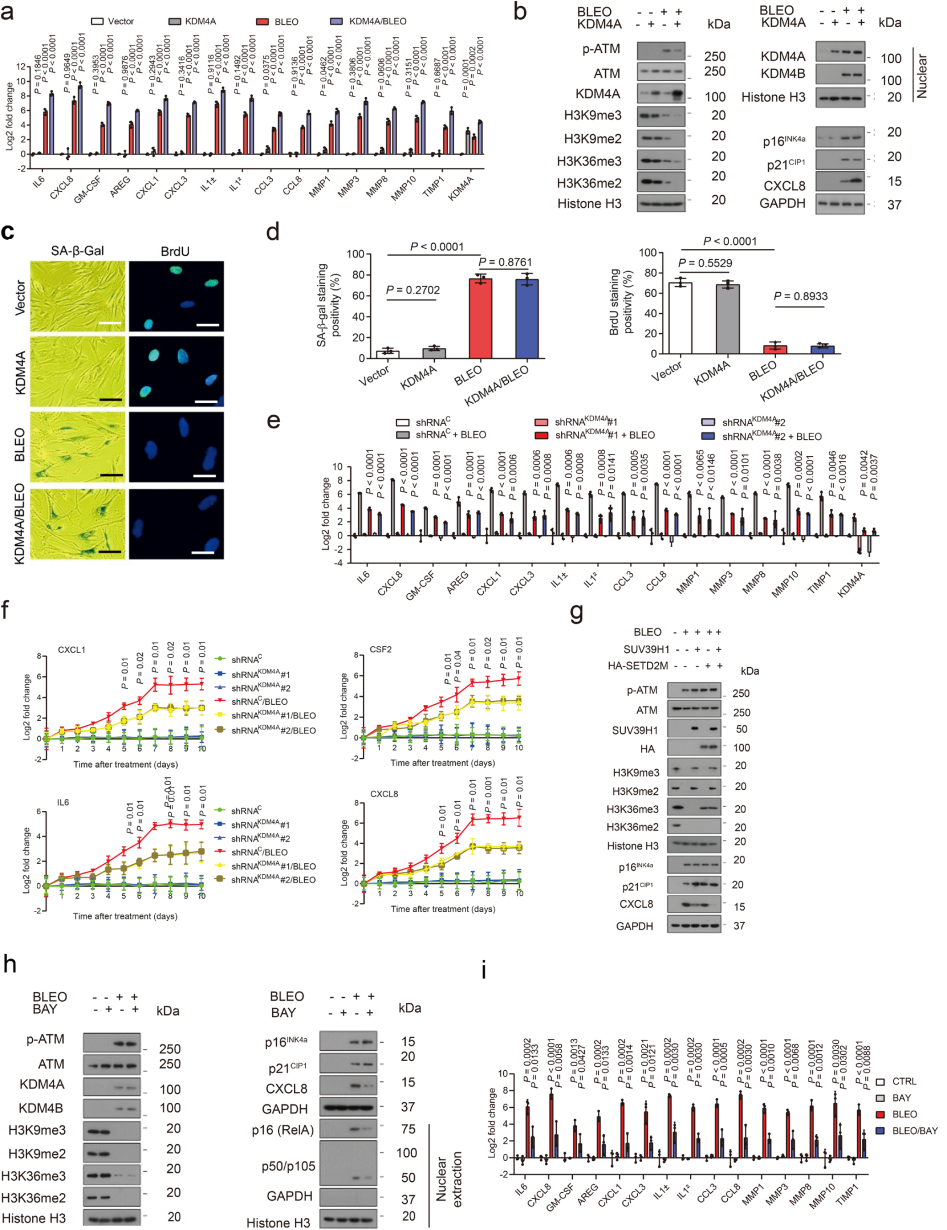
SASP expression is enhanced but H3K9/H3K36 methylation is attenuated by the histone demethylase KDM4A. (a) Quantitative assessment of SASP expression. PSC27 cells were transduced with a lentiviral construct encoding human KDM4A and/or exposed to BLEO before collection for expression assays. Signals were normalized to control cells (transduced with empty vector and untreated). (b) Immunoblot assay of DDR signaling, H3K9/H3K36 methylation, and SASP expression in cells processed in different ways, as described in (a). GPADH, loading control. Chromatin fractionation was performed to evaluate KDM4A/B in nuclei. Histone H3 was the nuclear lysate loading control. (c) Representative images of SA‐β‐gal and BrdU staining of PSC27 cells subjected to treatment as described in (a). Scale bar, 10 μm. (d) Comparative statistics of SA‐β‐gal and BrdU staining results of stromal cells in individual conditions of (a). (e) Expression profiling of hallmark SASP factors in PSC27 cell sublines transduced with lentiviral constructs encoding KDM4A‐specific shRNAs. Scrambled, shRNA control. Cells were subjected to vehicle or BLEO treatment before collection. (f) Expression curves of hallmark SASP factors in stromal cells treated in conditions as described in (e). Cells lysed at the indicated time points after BLEO damage. (g) Immunoblot assays of stromal cells transduced with lentiviral constructs encoding human SUV39H1 (full length), SETD2M (histone methylase domain SET, tagged with HA) or both. Cells were subjected to BLEO treatment after transduction, and lysates were collected 7–10 days later. GAPDH, loading control. (h) Immunoblot analysis of stromal cells treated with BLEO and/or Bay 11–7082 (BAY). Cell lysates were collected 7–10 days after treatment. Nuclear lysates were also prepared to assess nuclear translocation of representative NF‐κB subunits, p65 (RelA) and p50/p105. GAPDH, loading control. BAY (Bay 11–7082), an NF‐κB inhibitor. (i) Quantitative assessment of SASP expression. Cells were subjected to treatment(s) as described in (h). Signals were normalized to CTRL cells per gene. Data in all bar plots shown as mean ± SD and representative of three biological replicates. In (a, e, f, and i) *p* values were determined by two‐sided unpaired *t*‐tests and adjusted for multiple comparisons. Data in (b, c, g, and h) are representative of three biological replicates. GM‐CSF, granulocyte‐macrophage colony‐stimulating factor.

We next interrogated the relevance of KDM4A/B to SASP development in senescent cells. Data from cell transduction assays showed that KDM4A overexpression did not change the basal level of SASP factors, while after DNA damage, the expression of the majority of SASP factors was further enhanced by ectopic KDM4A (Figure 4a). This tendency was validated at the protein level by immunoblots, as reflected by enhanced CXCL8 expression in senescent cells, a process accompanied by further reduced H3K9/H3K36 methylation (trimethylated and dimethylated forms) intensities upon exogenous KDM4A expression (Figure 4b).

As KDM4A/B share a similar domain architecture including two PHD and two TUDOR domains, we speculated that the influence of KDM4A on the SASP can be basically phenocopied upon KDM4B transduction. This hypothesis was substantiated by evidence from a similar set of transcript and protein data (Figure 4a,b). It is noticeable that there was a pronounced nuclear translocation of both KDM4A and KDM4B after genotoxic treatment, although ectopic expression of A and B enhanced the nuclear amount of the transduced factor per se, respectively (Figure 4b, Figure S6b). When cells were exposed to Chaetocin, a histone methyltransferase inhibitor against SUV39H1, which preferentially catalyzes H3K9 methylation (H3K9me2/me3), the data partially resembled those of KDM4A/B expression (Figure S6c). Interestingly, however, expression of p16^INK4a^ and p21^CIP1^, the key CDK inhibitors indicative of cellular senescence, remained largely unaffected upon transgenic expression of KDM4A/B, suggesting that cell cycle arrest, or alternatively, cellular senescence, is likely not regulated by these epigenetic factors. To validate this assumption, cellular senescence and cell cycle arrest assays with senescence‐associated beta‐galactosidase (SA‐β‐gal) staining and bromodeoxyuridine (BrdU) incorporation assays were performed, respectively. The resulting data supported that expression of neither KDM4A nor KDM4B was sufficient to influence cellular senescence or replication (Figure 4c,d, Figure S6d,e).

We then selectively eliminated these two factors from stromal cells with short hairpin RNA (shRNA), after which cells were subjected to BLEO‐induced senescence. Again, removal of KDM4A/B abrogated SASP expression (Figure 4e, Figure S6f,g). We evaluated the induction pattern of several hallmark SASP factors including CXCL8, CSF2, CXCL1, and IL‐6 by graphing time‐dependent curves and noticed the fold change of these factors was significantly restricted upon KDM4A/B depletion, starting from senescence induction (Figure 4f). Interestingly, however, the development of cellular senescence per se was not affected upon knockdown of KDM4A/B (Figure S6h,i).

Next, we queried the influence of histone methyltransferases SUV39H1 and SETD2, the latter specifically responsible for trimethylation of the H3K36 site (H3K36me3), on cellular senescence and the SASP. To this end, we transduced the full‐length human SUV39H1 and the methyltransferase domain of human SETD2 (SETD2M, which encodes amino acids 946–1738 as the SET domain and mediates SETD2 catalytic function) (Chen et al. 2017), individually or simultaneously before genotoxic treatment. Immunoblots indicated that ectopic expression of these epigenetic factors failed to abrogate cellular senescence, with p21^CIP1^ expression even slightly enhanced (Figure 4g). Importantly, however, each of these methyltransferases was able to reduce the SASP expression, as evidenced by diminished CXCL8 signals, although concurrent transduction of SUV39H1 and SETD2M generated the most dramatic effect. Thus, epigenetic modification of certain sites of chromatin histone H3.2, specifically trimethylation of H3K9 and H3K36, holds the potential to restrain the intensity of the secretome while sustaining cell cycle arrest.

To further dissect the role of KDM4A/B in SASP development, we treated cells with Bay 11–7082 to block nuclear factor‐kappa B (NF‐κB), a master regulator of the SASP (Chien et al. 2011). Of note, the expression of the SASP was markedly suppressed in senescent cells upon NF‐κB blockade, while KDM4A/B induction persisted (Figure 4h,i). The data suggest that histone H3 demethylation mediated by KDM4A/B is a prerequisite, but not the sole driver of the SASP, full development of which requires functional activation of transcriptional regulators including but not limited to the canonical NF‐κB complex.

### 2.5 Targeting the Demethylase Activity of KDM4 Restrains the Senescence‐Associated Secretory Phenotype

**FIGURE 5:**
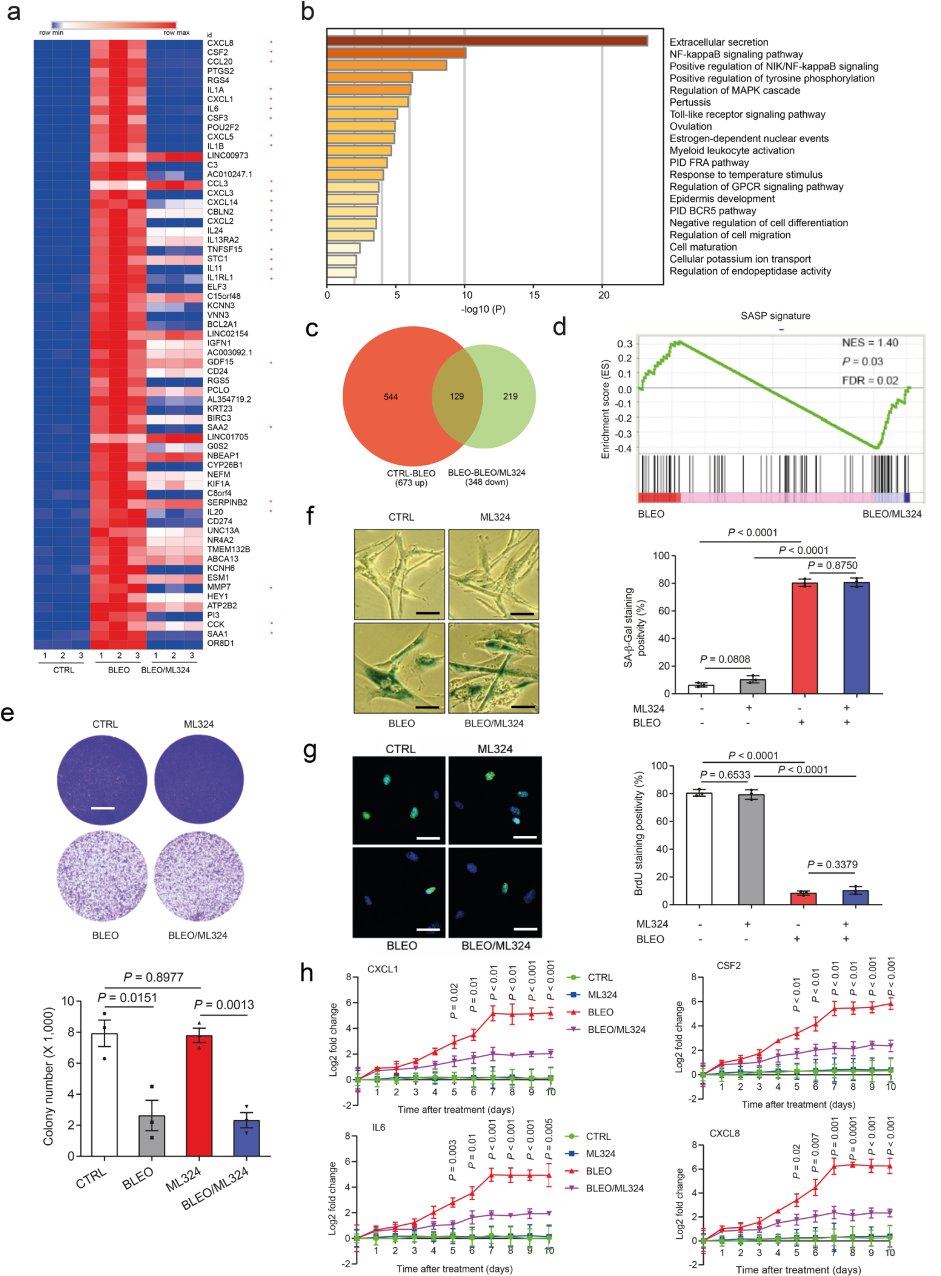
Targeting KDM4 with a small molecule inhibitor interferes with the SASP but not cell growth or cell cycle arrest. (a) Heat map depicting the influence of DNA damage and ML324, a selective chemical inhibitor of KDM4, on the transcriptomic expression profile of PSC27 cells. Genes sorted by expression fold change when comparing between cells treated by CTRL versus BLEO (highest on top). Asterisks indicate canonical SASP factors affected by ML324. (b) Graphic visualization of pathways by GO profiling. Significantly enriched genes were those downregulated and sorted according to their fold change when senescent cells were exposed to ML324. GPCR, G‐protein‐coupled receptors; BCR, B cell receptor; PID, pelvic inflammatory disease; FRA, Fos‐related antigen. (c) Venn diagram representation of genes upregulated by BLEO (673, relative to CTRL) and downregulated genes by ML324 (348, in relative to BLEO). (d) Gene‐set enrichment analysis profiling of gene expression with significant enrichment scores showing a SASP‐specific signature in BLEO/ML324 co‐treated cells compared with BLEO only‐treated cells. FDR, false discovery rate; NES, normalized enrichment score. (e) In vitro colony formation assay of cells exposed to BLEO and/or ML324 treatment. The upper images are representative images of crystal violet staining; the graph in the lower panel indicates comparative statistics. Scale bar, 200 μm. *p* values were determined by two‐sided unpaired *t*‐tests and adjusted for multiple comparisons. (f) SA‐β‐gal staining of cells after treatment by BLEO and/or ML324. Left, representative images. Scale bar, 10 μm. Right, statistics. (g) BrdU staining of cells treated as described in (e). Scale bar, 10 μm. (h) Time course expression of a subset of SASP factors (CXCL8, CSF2, CXCL1, and IL‐6). Cells were subjected to BLEO and/or ML324 treatment. Data in all bar plots are shown as mean ± SD and representative of three biological replicates. In (d) the statistical significance was calculated using one‐way ANOVA with Tukey's post hoc comparison. In (e) (bottom panel), (f and g (right) and h) *p* values were determined by two‐sided unpaired *t*‐tests and adjusted for multiple comparisons.

Given the critical role of KDM4A/B in SASP development, we interrogated whether pharmacologically targeting their demethylase activities can effectively control the SASP in senescent cells. To address this, we employed ML324, a small molecule compound selectively inhibiting the activities of the KDM4 family (Duan et al. 2019). RNA‐seq data suggested that the expression of the majority of SASP factors in senescent cells was markedly diminished upon ML324 treatment, including but not limited to CXCL8, CSF2, CCL20, IL1A, CXCL1, and IL6 (Figure 5a). Among genes significantly upregulated upon cellular senescence, a considerable portion showed reduced expression when cells were exposed to ML324, although some SASP‐unrelated factors were also affected by this agent, such as RGS4, POU2F2, and C3 (Figure 5a). Gene Ontology (GO) analysis showed that the most suppressed pathways and biological processes by ML324 were correlated with extracellular secretion, NF‐κB signaling, receptor tyrosine phosphorylation, and the MAPK cascade, activities generally characteristic of canonical SASP factors (Figure 5b). Further bioinformatics validated an overlapping zone comprising 129 genes between those upregulated upon cellular senescence and those downregulated upon ML324‐mediated KDM4 inhibition, the latter evidenced by rescued methylation of H3K9/H3K36 sites (trimethylated and dimethylated forms) and accompanied by diminished CXCL8 expression (Figure 5c, Figure S7a). Gene‐set enrichment analysis confirmed that restraining KDM4A/B activities effectively dampened the SASP and disturbed NF‐κB activity (Figure 5d, Figure S7b); however, ML324 treatment did not interfere with cell colony formation or cell growth arrest (Figure 5e, Figure S7c). Interestingly, a number of genes whose expression declined in senescent cells appeared to be upregulated upon cell exposure to ML324 (Figure S7d), suggesting that KDM4A/B inhibition alters the expression of a spectrum of genes, among which are those encoding the SASP factors, and affected genes are not limited to those upregulated in senescent cells. Although the mechanism of ML324 in reversing the expression of many SASP‐canonical and a handful of SASP‐noncanonical factors remains unclear, further work to define the impact of KDM4 functional deficiency on the transcriptome‐wide expression profile of senescent cells is warranted.

In vitro assays including SA‐β‐gal staining and BrdU incorporation suggested that ML324 did not affect senescence or cell cycle arrest, consistent with findings that KDM4A/B are not essential for the maintenance of senescence per se (Figure 5f,g). Gene expression curves substantiated that ML324 significantly restricted expression of SASP factors, as exemplified by the cases of CXCL1, CSF2, IL‐6, and CXCL8, with the efficacy evident during cellular senescence induction (Figure 5h). To define further the ML324‐generated SASP‐targeting consequence, specifically in a virtual microenvironment, we used a co‐culture system that involves treatment of cancer cells with stromal cell‐derived conditioned media. Notably, the capacity for proliferation, migration, and invasion of PC3, DU145, LNCaP, and M12, typical PCa cell lines sharing the same organ origin as the PSC27 stromal line, namely human prostate, considerably decreased upon ML324‐mediated treatment of stromal cells (Figure S7e–g). Further data from experimental assays performed with gene‐specific shRNA‐mediated knockdown of KDM4A/B largely confirmed observations from ML324 treatment. More importantly, resistance of PCa cells to MIT, a chemotherapeutic agent frequently administered to patients with PCa in cancer clinics, was significantly reduced when stromal cells were subjected to KDM4 targeting (Figure S7h,i). We experimentally assessed the influence of stromal KDM4 deficiency on lung cancer cell behaviors and found similar results (Figure S8a–e). Thus, suppression of the demethylase activity of KDM4 with a small molecule inhibitor or shRNA can retard SASP development without compromising senescence, causing diminished potential of the SASP to promote cancer progression.

### 2.6 Chromatin Remodeling Supports SASP Development Which Is Uncoupled From Senescence Upon KDM4 Targeting

The state of chromatin dictates vital cellular processes such as DNA repair and gene expression, while accessible chromatin marks can decorate regulatory sequences including enhancers, promoters, and locus‐control regions to cooperatively regulate gene expression (Klemm et al. 2019). We next sought to determine the possibility and significance of chromatin structure alterations in supporting SASP development.

**FIGURE 6:**
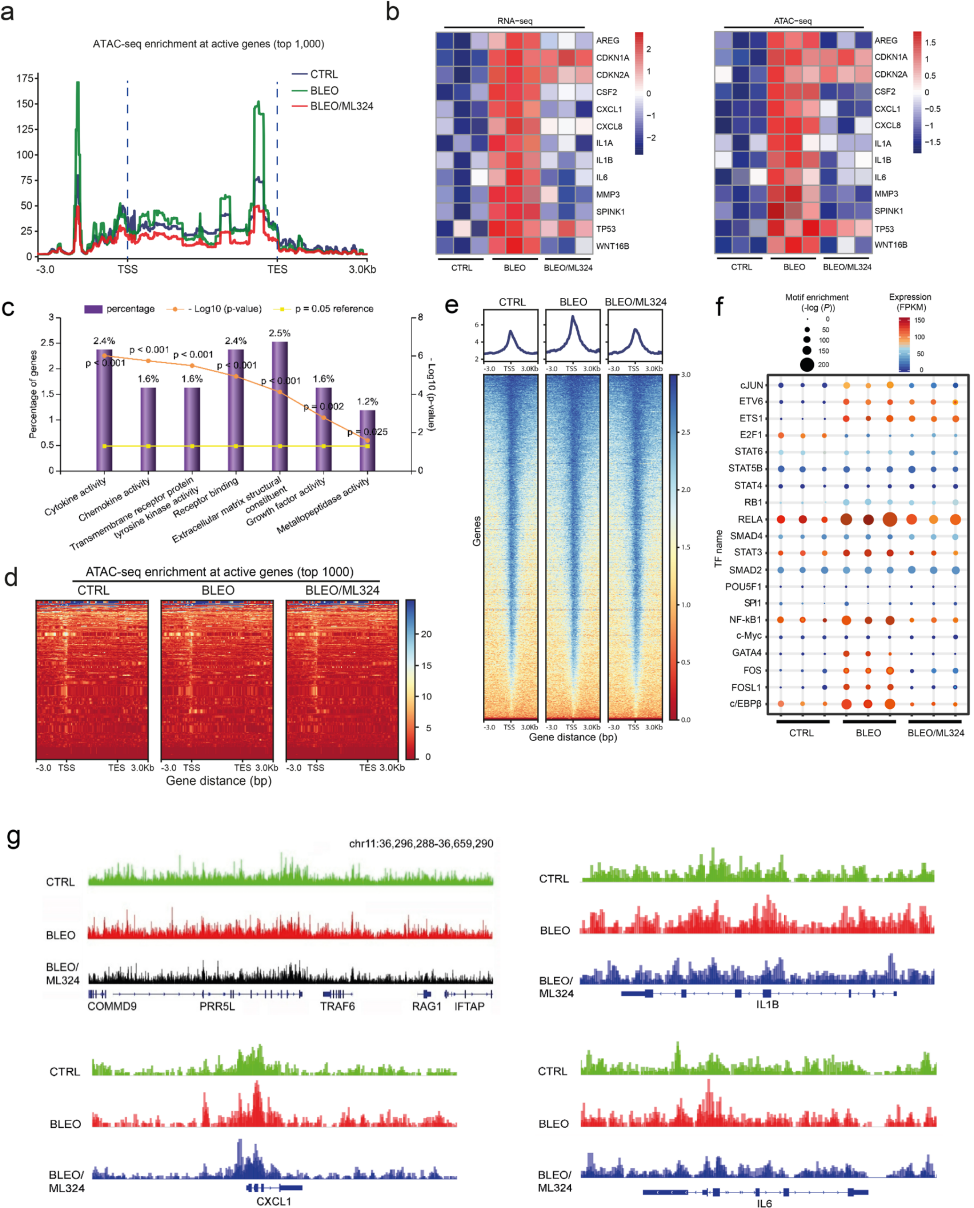
Accessible chromatin landscape in senescent cells and ML324‐mediated suppression. (a) The average level of ATAC‐seq enrichment (normalized) for the top 1000 most active genes between proliferating cells, SEN cells (bleomycin‐induced) and bleomycin/ML324 co‐treated cells (marked as CTRL, BLEO and BLEO/ML324, respectively). (b) Heat maps showing the expression in fragments per kilobase of transcript per million mapped reads (FPKM) of the SASP hallmark genes (RNA‐seq) and the assessable chromatin enrichment in reads per kilobase of bin per million reads sequenced (RPKM; ATAC–seq) at their promoters (TSS ± 2.5 kb). Example genes are listed alongside each type of heat map. (c) GO analysis results for gene classes of significant expression fold change in proliferating versus senescent cells, and inhibited substantially upon ML324 treatment. Percentage of gene number among all upregulated genes in SEN cells and log10 of *p* value per class presented, with *p* < 0.05 (two‐sided unpaired test) as the threshold for significance. (d) Heat maps showing ATAC‐seq enrichment of peaks near the accessible promoters (3.0‐kb upstream of the TSS and downstream of the TES per gene) in each of the assayed samples. Enrichment signals were collected for all active TSSs and TESs, which were assorted by cap analysis of gene expression values, with peaks defined by hierarchical clustering. The top 1000 genes of enrichment signals were selected for analysis. (e) Heat maps depicting ATAC‐seq enrichment of peaks near the accessible promoters (3.0‐kb upstream of the TSS and downstream of the TES per gene) present in each of the assayed samples. The whole genomic range was evaluated for each sample. (f) TF motifs identified from distal ATAC‐seq peaks in each of the group's samples. Only TFs with detectable expression (FPKM ≥ 5) and a motif enrichment *p* value < 1 × 10^−10^ in each sample were included. (g) The UCSC browser views show enrichment of ATAC‐seq signals at the promoters of several SASP‐unrelated genes (*COMMD9*, *PRR5L*, *TRAF6*, *RAG1* and *IFTAP*) in contrast to those at the promotes of representative SASP factors (CXCL1, IL‐1β and IL‐6).

We first investigated whether the accessible chromatin landscape of senescent cells differs from that of their proliferating counterparts with the assay for transposase‐accessible chromatin with high‐throughput sequencing (ATAC‐seq), a well‐established epigenomic strategy. Surprisingly, we observed unusually strong ATAC‐seq signals upstream (~0.5 kb) of transcription end sites (TESs) of transcriptionally active genes in senescent cells (Figure 6a). Despite a sharp increase in signals at approximately 2.0 kb upstream of transcription start sites (TSSs), the difference between senescent and proliferating cells seems to be limited at proximal regions of TSSs. However, upon ML324 treatment, we observed a remarkable decrease of enrichment signals at both TSSs and TESs of senescent cells (Figure 6a). The TES‐open chromatin likely reflects binding of factors involved in transcriptional termination, while these sites may alternatively function as enhancers promoting high‐level transcription of biologically essential genes (Wu et al. 2016). Our data suggest that open chromatin can be found both at promoters and near TES regions of transcriptionally active genes in senescent cells.

Subsequent results from analysis mapping of ATAC‐seq and RNA‐seq outputs indicate that transcriptional levels of a set of typical SASP factors were intimately correlated with intensities of their promoter ATAC‐seq signals, although those of CDK inhibitors (e.g., CDKN1A, CDKN2A, and TP53) remained essentially unchanged (Figure 6b). We noticed most genes upregulated upon senescence but downregulated upon ML324 treatment have implications in cytokine, chemokine, transmembrane receptor binding, extracellular matrix structure, growth factor, and metalloproteinase‐associated activities (Figure 6c).

Enrichment‐based heatmaps illustrating either the most active 1000 genes (~3.0 kb upstream of the TSS and downstream of the TES per gene) or whole transcriptomics, displayed striking difference between control and senescent cells, as well as between senescent cells exposed to vehicle and to ML324 (Figure 6d,e, Figure S9a). As enhancers represent hotspot sites for transcription factor (TF) binding (Buecker and Wysocka 2012), we reasoned that distal ATAC‐seq peaks may harbor motifs for TFs regulating senescence and/or related phenotypes. Using the motif analysis program HOMER, we extracted the binding motifs for an array of TFs enriched in distal peaks found in either of the three experimental conditions of PSC27 cells (Figure 6f). Some of these TFs, including AP‐1 family members (cJUN, FOS and FOSL1), RELA (p65 of NF‐κB), STAT3, NF‐κB1 (p50/p105 of NF‐κB), GATA4, and c/EBPβ, factors reported to be correlated with SASP (Chen et al. 2018; Huggins et al. 2013; Kang et al. 2015; Nacarelli et al. 2019; Toso et al. 2014), clearly showed a ‘senescence‐up and ML324‐down’ pattern. However, certain TFs such as ETV6, ETS1 and RB1, displayed enhanced binding capacity in senescent cells, a tendency seemingly not affected upon KDM4 suppression. Other TFs such as E2F1 manifested reduced activity in senescent cells and were not rescued by ML324. Together, senescent cells exhibited distinct landscapes for distal ATAC‐seq peaks, while significant gene expression is correlated with a handful of ‘master TFs’ regulating the senescence‐specific circuitry and potently reset upon KDM4 deficiency induced by ML324. In addition, we noticed there was a set of SASP‐unrelated genes whose ATAC‐seq signals were substantially decreased in senescent cells but apparently reversed upon cell exposure to ML324 (Figure S9b), suggesting the influence of KDM4 inhibition may not be exclusively limited to chromatin accessibility changes underlying SASP expression.

We then performed footprinting analysis of accessible chromatin for specific genes. The reproducibility between biological replicates of ATAC‐seq was first confirmed with a small group of genes, including *COMMD9*, *PRR5L*, *TRAF6*, *RAG1*, and *IFTAP*, which are presumably not correlated with senescence (Figure S9c). We assessed the data from allelic ATAC‐seq enrichment assays and uncovered open genomic regions of typical SASP factors, including IL1β, CXCL1, IL6, AREG, SPINK1, MMP3, and WNT16B, in senescent cells, but with a markedly reduced accessibility upon KDM4 suppression (Figure 6g, Figure S9d). In contrast, the chromatin openness of genes not correlated with senescence remained essentially unaltered, such as *COMMD9*, *PRR5L*, and *TRAF6* (Figure 6g). CDKN2A and TP53 were selected for parallel analysis, showing enhanced accessibility in senescent cells but largely sustained upon ML324 exposure (Figure S9e). Thus, chromatin accessibility and transcriptional expression are intimately correlated for SASP hallmark factors, while active regulatory elements are accessible by the cell's expression machinery, such as a special set of key TFs.

Having established the correlation between targeted chromatin openness and pro‐inflammatory gene expression upon senescence, we further performed unbiased genome‐wide studies. To streamline genomic location analysis in combination with expression assays, we first mapped occupancy patterns of H3K9me3 and H3K36me3 via chromatin IP combined with high‐throughput sequencing (ChIP‐seq). In contrast to proliferating counterparts, senescent cells exhibited markedly reduced peak numbers for both H3K9me3 and H3K36me3. However, these changes were essentially counteracted upon KDM4 suppression, although the total number and distribution of peaks associated with H3K9me3 and H3K36me3 differed in each situation (Figure S10a). Notably, the differential distribution pattern of H3K9me3/H3K36me3 between proliferating and senescent cells is analogous to that derived from the comparison of senescent cells and those exposed to ML324 treatment (Figure S10b). Further exploration of the output data discovered a substantial number of genes whose association with H3K9me3/H3K36me3 was essentially modified upon senescence but largely reversed by ML324, suggesting the pivotal role of KDM4 in epigenetic remodeling (Figure S10c, Tables S4 and S5).

We further observed reduced signal intensities of H3K9me3 at the proximal TSS sites of typical SASP factors (excluding IL6 and AREG, not enriched with H3K9me3 even at naive) upon cellular senescence, a tendency largely counteracted in the presence of ML324 (Figure S11a; see Tables S6–S9 for genome‐wide peak calling and differential expression mapping). Of note, for some SASP factors such as AREG, MMP3, and WNT16, H3K36me3 displayed even increased enrichment on gene bodies, despite its globally reduced peak number and signal intensity upon cellular senescence (Figure S11a, Figure 1f). The feature of H3K36me3, a mark of active chromatin regions, partially resembles that of H3K27ac, the signals of which can be globally reduced but locally increased at active enhancers and TSSs during organismal aging (Adelman et al. 2019; Benayoun et al. 2019).

We next performed ChIP‐qPCRs to probe signals associated with KDM4A/B demethylases. Due to the lack of ChIP‐grade antibodies for KDM4B, we focused on experimental assays for KDM4A, data of which essentially supported observations from ATAC‐seq mapping and RNA‐seq profiling (Figure S11b).

### 2.7 Therapeutically Targeting KDM4 Minimizes Chemoresistance and Enhances Preclinical Index

Given the prominent role of KDM4 in the development of cellular senescence‐associated phenotypes, specifically the SASP, we next reasoned the potential of selectively harnessing this target to improve therapeutic efficacy of age‐related disorders. As cancer represents the leading cause of age‐standardized incidence and premature mortality in the global range and is adversely correlated with the impact of senescent cells (Basisty et al. 2020; Ward et al. 2019), we chose the TME as a pathological entity for subsequent in vivo manipulation.

**FIGURE 7:**
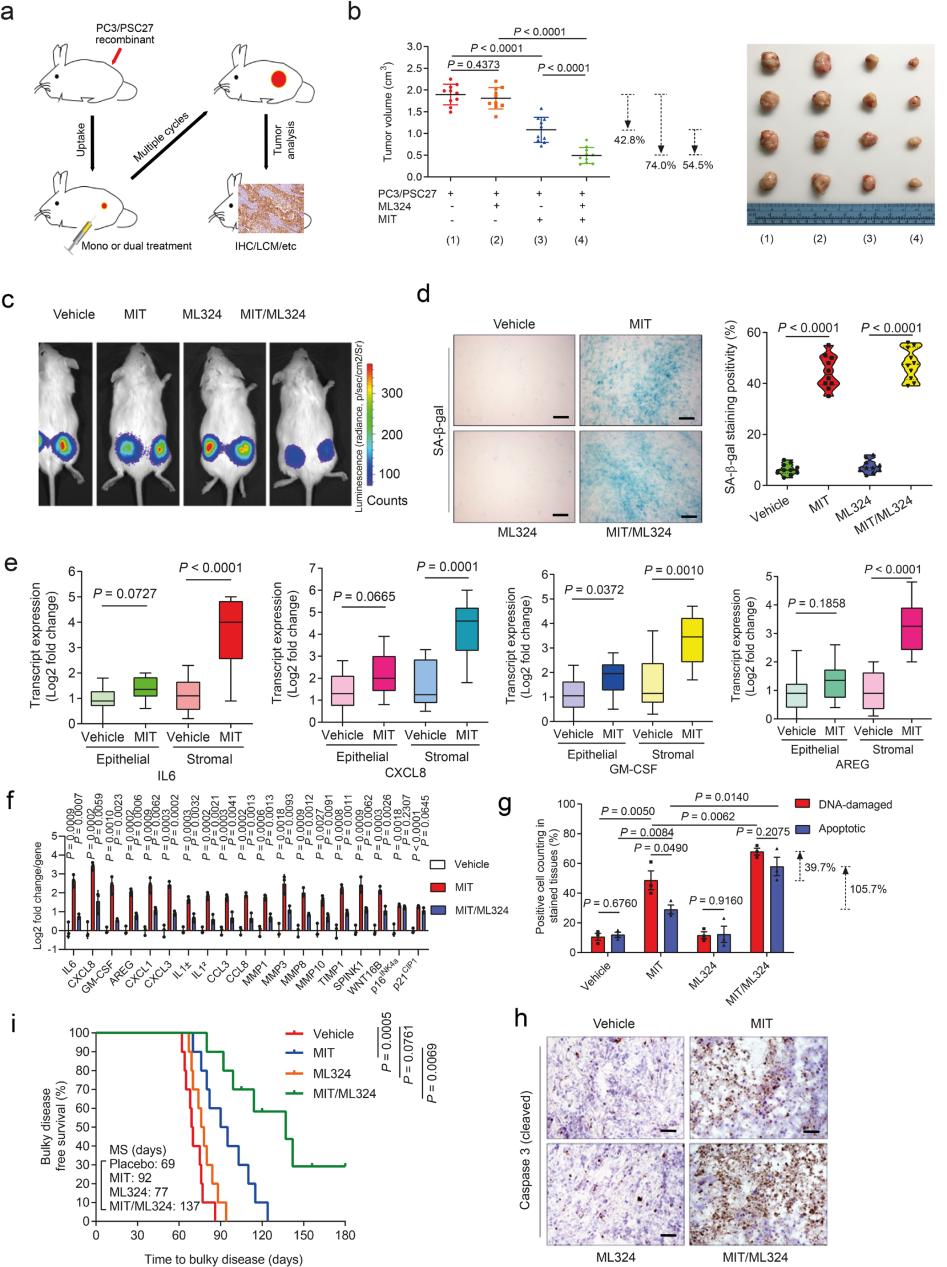
Therapeutically targeting KDM4 in the damaged TME diminishes cancer resistance conferred by senescent stroma. (a) Illustrative diagram for preclinical treatment of NOD/SCID mice. Two weeks after subcutaneous implantation and in vivo uptake of tissue recombinants, animals received either single (mono) or combinational (dual) agents administered as metronomic treatments. (b) Statistical profiling of tumor end volumes. PC3 cells were xenografted alone or together with PSC27 cells. MIT was administered to induce tumor regression, alone or together with ML324. Right, representative images of tumors. c, Representative BLI of PC3/PSC27 tumor‐bearing animals. Digital signals were proportional to in vivo luciferase activities measured by an IVIS device. (d) Comparative imaging of in vivo senescence of tumor tissues by SA‐β‐gal staining. Scale bars, 200 μm. Right, violin plot of positivity statistics. (e), Transcript assessment of in vivo expression of several canonical SASP factors in stromal cells isolated from tumors of NOD/SCID mice. Statistics were performed with a two‐sided Mann–Whitney *U*‐test, with upper and lower hinges representing the 25th and 75th percentiles. Horizontal bars show the median value, and whiskers extend to the values no further than 1.5 times the interquartile range from either the upper or lower hinge. (f) Quantitative appraisal of SASP factor expression and senescence marker expression in stromal cells isolated from tumor tissues of animals. Signals for each factor were normalized to the vehicle‐treated group. (g) Statistical assessment of DNA damage and apoptosis in preclinical biospecimens. Values are the percentage of cells positively stained with antibodies to γ‐H2AX or caspase 3 (cleaved). (h) Representative histological images of caspase 3 (cleaved) in tumors at the end of therapeutic regimes. Scale bar, 100 μm. (i) Survival appraisal of mice killed upon development of advanced bulky diseases. Survival duration was calculated from the time of tissue recombinant injection until the day of death. Data were analyzed by a two‐sided log‐rank (Mantel‐Cox) test. Data in all bar plots are the mean ± SD and representative of three biological replicates. In (b (left), d (right), f and g) *p* values were from a two‐sided unpaired *t*‐test and adjusted for multiple comparisons. Data in (h) are representative of three biological replicates.

First, we generated tissue recombinants by admixing stromal cells (PSC27) with cancer cells (PC3) at a pre‐optimized ratio before subcutaneous implantation in experimental mice with non‐obese diabetes and severe combined immunodeficiency (NOD/SCID). To closely simulate clinical conditions, we designed a preclinical regimen incorporating the genotoxic agent MIT and/or the KDM4‐specific inhibitor ML324 (Figure 7a). Two weeks after implantation, a single dose of therapeutic agent or placebo (vehicle) was delivered on the first day of the 3rd, 5th, and 7th week until the end of the 8‐week regimen (Figure S12a). To determine the influence of therapeutic agents on the growth of tumors lacking stromal cells, we first treated mice xenografted with only PC3. MIT treatment significantly affected tumorigenesis, while ML324 failed to provide therapeutic benefit either alone or in combination with MIT (Figure S12b,c). In contrast, we found MIT administration alone caused a significant reduction in the size of PC3/PSC27 tumors, while the addition of ML324 further decreased tumor mass (54.5%), allowing an overall shrinkage of 74.0% (Figure 7b, Figure S12d). Bioluminescence imaging (BLI) of xenografts comprising PC3 cells stably expressing luciferase (PC3‐luc) and PSC27 cells excluded the potential metastasis of cancer cells from the primary sites, with signal intensities largely consistent with tumor growth patterns observed in PC3/PSC27 animals (Figure 7c). The data suggest that classic chemotherapy combined with a KDM4‐targeting agent can induce tumor regression with an index significantly higher than that of chemotherapy alone. To exclude the off‐target effects of ML324, we alternatively generated tumors composed of cancer cells and stromal cells, which were pre‐engineered to deplete KDM4A/B before they were xenografted to animals, and the resulting data largely confirmed the benefit of targeting KDM4A/B in tumor intervention (Figure S12e).

We observed a considerable percentage of senescent cells in the foci of mice treated with agents involving MIT, which presumably resulted from genotoxicity‐induced tissue damage (Figure 7d). LCM‐supported transcript evaluation revealed significantly increased expression of SASP factors including but not limited to IL6, CXCL8, SPINK1, WNT16B, IL1α, MMP3, and GM‐CSF, a tendency accompanied by remarkable upregulation of p16^INK4a^ and p21^CIP1^ (Figure 7e, Figure S12f,g). However, these changes appeared generally limited to stromal cells, rather than their adjacent epithelial counterparts. We further assessed the in situ inducibility of KDM4A/B in the stromal compartment of tumor samples, and noticed their substantial expression in animals experiencing MIT‐involved treatments (Figure S13a–d), thus consolidating our in vitro findings (Figure 3a,b). Signals of H3K9me3 and H3K36me3 were significantly reduced in stromal cells by MIT, a tendency reversed upon MIT/ML324 co‐treatment (Figure S13e). Together, these findings suggest the incidence of in vivo cellular senescence accompanied by development of a typical SASP, expression of KDM4A/B, and decreased histone H3 methylation at K9/K36, a side effect induced by genotoxicity during chemotherapeutic intervention and mainly observed in the benign compartments of the TME.

We further found that the genotoxic treatment induced a full spectrum of SASP expression in the damaged TME, as evidenced by the pattern observed in stromal cells of tumor foci but markedly counteracted by ML324 (Figure S13f). In contrast to diverse SASP components, senescence markers such as p16^INK4a^ and p21^CIP1^ were upregulated upon chemotherapy, but remained largely unchanged when ML324 was delivered (Figure 7f). Thus, preclinical data substantiate our in vitro findings that KDM4 inhibition retards SASP development, but not cellular senescence, a phenomenon correlated with KDM4‐mediated histone H3 demethylation, which enables chromatin reorganization.

To explore the mechanism underlying MIT‐induced cancer resistance, we dissected tumors from animals treated by different agents 7 days after treatment, a time point before the emergence of resistant colonies. MIT administration caused remarkable DNA damage and apoptosis, a pattern not observed in tumors dissected from ML324‐treated animals (Figure 7g). Although ML324 alone induced neither typical DDR nor substantial cell death, the combination of MIT with ML324 significantly enhanced both DNA damage and cell apoptosis (Figure 7g). Histological appraisal against caspase 3 (cleaved), a typical biomarker of cell apoptosis, confirmed these different responses (Figure 7h).

Given the remarkable benefit of targeting KDM4 in a treatment‐damaged TME, we next sought to assess animal survival in a time‐extended manner. Development of a bulky disease is considered once tumor burden becomes prominent (e.g., volume > 2000 mm^3^) (Melisi et al. 2011; Zhang et al. 2018). Mice receiving MIT/ML324 co‐treatment exhibited the most prolonged median survival duration, acquiring a minimum of 50% longer survival than the group treated with MIT alone (Figure 7i). However, ML324 alone did not provide significant benefit, conferring only marginal survival elongation (Figure 7i). Thus, KDM4‐targeting changes neither tumor growth nor animal survival, while MIT/ML324 co‐treatment significantly improves both parameters.

More importantly, experimental data suggest that dual administration was well tolerated by mice, as no significant perturbations in body weight, renal function (creatinine and urea) or liver integrity (alkaline phosphatase and alanine transaminase) were observed (Figure S14a,b). Therapeutic agents did not significantly interfere with organ metabolism, the immune system, or tissue homeostasis, even in immunocompetent animals (C57BL/6 strain; Figure S14c–e). These results together support that ML324, a typical KDM4 inhibitor, combined with conventional chemotherapy holds the potential to promote tumor response without generating severe and systemic cytotoxicity in critical organs.

To expand, we further tested the treatment efficacy and safety with several additional mouse groups. Data from animals such as those involving LNCaP (androgen receptor positive) or 22Rv1 (hormone refractory), alternative PCa cell lines (Figure S15a–h), as well as those carrying lung cancer cells (A549; Figures S16a–j and S17a,b), consistently substantiated the feasibility of our regimen in restraining tumor development and demonstrated the translational value via several lines of preclinical trials.

## 3 Discussion

Aging is a degenerative process correlated with epigenetic dysregulation, which disrupts gene expression patterns and compromises tissue function and regenerative capacity (Lu et al. 2020). Senescent cells accumulate in diverse organ types with age and progressively reside in damaged or dysfunctional tissues. Besides exiting the cell cycle, senescent cells undergo multiple phenotypic alterations such as morphological abnormality, metabolic reprogramming, and chromatin reorganization (Herranz and Gil 2018). They actively synthesize and release a large number of soluble factors, collectively termed the SASP, which mediate most of the senescence‐associated cell‐nonautonomous effects (Childs et al. 2018; Khosla et al. 2020). Senescent cells have attracted increasing attention as a potent therapeutic target for age‐related diseases, including cancer, a leading cause of morbidity and mortality in the elderly (Song et al. 2020). Considering the beneficial effects of cellular senescence in special circumstances such as those in tissue repair, wound healing, and embryonic development (Gorgoulis et al. 2019; Sun et al. 2018), however, it may be more advantageous to specifically diminish the SASP, rather than the radical elimination of these cells from tissue microenvironments in many situations. In this study, we unraveled an epigenetic mechanism functionally supporting SASP development and defined the key target KDM4 to manipulate senescent cells by dampening the SASP while retaining cell cycle arrest. We further established an effective strategy to improve therapeutic outcomes of age‐related pathologies, specifically cancer, by integrating classic chemotherapy and KDM4‐specific agents as a solution guided by advanced concepts.

**FIGURE 8:**
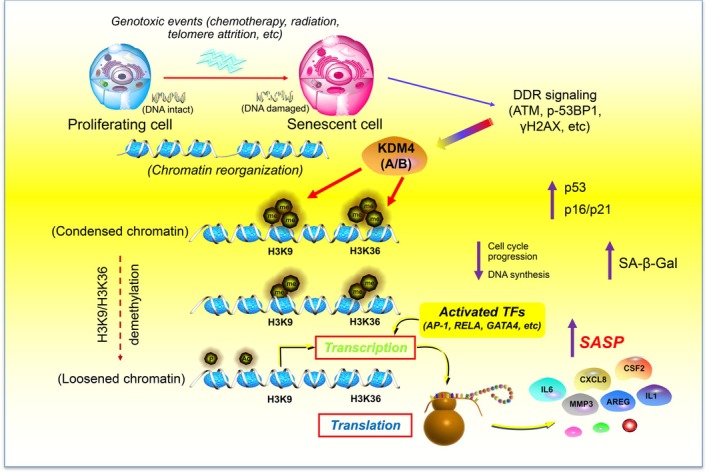
Working model depicting epigenomic reprogramming and KDM4‐mediated histone demethylation (H3K9/H3K36), events enabling SASP expression in senescent cells. In genotoxic settings, senescent cells undergo irreversible DNA damage, which triggers enhanced DDR signaling. The chromatin accessibility landscape is remodeled, with a set of activated TFs physically binding to the enhancers and promoters of senescence‐associated genes, including those encoding SASP factors. There is a strong concordance of clustering schemes and a close functional linkage between chromatin accessibility and transcriptional output. Future efforts to combine genome and epigenome sequencing, as well as to generate maps of chromosome conformation, will pave the way to tackle the non‐coding genome in senescent cells. Importantly, technological pipelines demonstrating three‐dimensional epigenomes to allow identification of distinct modes of epigenetic regulation, and studies revealing dynamic engagement of key molecules including, but not limited to, KDM4 upon the onset of global reconfiguration of chromatin and transcription machineries for genome activation, hold the potential to precisely define the epigenetic landscape of senescent cells.

Our study not only featured the chromatin landscapes of senescent cells, but also allowed genome‐wide identification of regulatory circuitry during epigenomic remodeling, an event that occurs upon cellular senescence (Figure 8). Data from ATAC‐seq and RNA‐seq suggest that chromatin accessibility changes underlie key aspects of gene regulation, with transposable elements frequently enriched at distal regions of the gene promoter in senescent cells. However, data from H3K9me3 and H3K36me3 ChIP‐seq support that changes in these histone modifications also occur at proximal TSS sites. Future efforts to map these interactions and analyze the genome‐wide three‐dimensional spatial structure of chromatin, such as high‐throughput chromosome conformation capture (Hi‐C) and Hi‐C chromatin immunoprecipitation (Hi‐ChIP), will allow the definition of global genome topology of senescent cells. As interactions between promoters/enhancers and chromatin at certain loci contribute to enhanced transcription of senescence phenotype‐associated genes, specifically those encoding SASP factors, advanced technologies such as chromatin interaction analysis by paired‐end tag sequencing (ChIA‐PET) will favor the identification of protein‐mediated promoter and enhancer interactions and enable genome‐wide studies of specific interactions between broad domains and super enhancers.

KDM4 is overexpressed or deregulated in multiple cancer types, cardiovascular diseases, and mental retardation as potential broad‐spectrum therapeutic targets (Lee et al. 2020). Deregulated KDM is associated with chromatin instability, tumor suppressor silencing, oncogene activation, hormone receptor binding, and downstream signaling (Lee et al. 2020). Despite extensive investments, however, there are few KDM4‐selective inhibitors that can be exploited, presumably due to the structural similarity of KDM members and their conserved properties of the active site (Kim et al. 2019). Precise definitions of the pathological functions of KDM4 will provide the foundation for the discovery of novel and potent inhibitors, which may be designed to target alternative sites. Based on The Cancer Genome Atlas (TCGA) data compiled from cohorts of individuals with cancer (Figure S18a–d), we further highlight KDM4 as a multifaceted target and provide proof‐of‐principle evidence that it can be exploited to minimize the pathological impact of a treatment‐damaged TME harboring myriad senescent cells, a therapeutic approach exemplified by preclinical trials.

## 4 Methods

### 4.1 Cell Lines, In Vitro Culture and Lentivirus

Human primary prostate stromal cell line PSC27 was kindly provided by P. Nelson (Fred Hutchinson Cancer Research Center) and maintained in PSC complete medium as described previously (Sun et al. 2012). PCa cell lines PC3, DU145, LNCaP, and 22Rv1 were from the American Type Culture Collection and routinely cultured in DMEM or RPMI 1640 (for 22Rv1; supplemented with 10% FBS plus 1% penicillin–streptomycin). M12, a neoplastic and metastatic PCa cell line, was a gift from S. Plymate (University of Washington) (Bae et al. 1998). Lung cancer cell lines A549, NCI‐H460, and NCI‐H1299 were from the Cell Bank of the Chinese Academy of Sciences and routinely cultured in DMEM with supplements, as described above. All cell lines were routinely tested for microbial contamination and authenticated with STR assays. No cell line is found on the list of known misidentified cell lines maintained by the International Cell Line Authentication Committee. Lentiviral particles were produced using Lipofectamine 2000 and a packaging kit (Thermal Scientific) based on the manufacturer's instructions. PSC27 infected with viruses encoding the puromycin resistance gene were selected using puromycin (1 μg mL^−1^) for 3 days.

### 4.2 Cancer Patient Recruitment and Clinical Studies

Chemotherapeutic administration involving genotoxic agents was performed for patients with primary PCa (clinical trial no. NCT03258320) by following the CONSORT 2010 Statement (updated guidelines for reporting parallel group randomized trials). Patients with a clinical stage ≥ I subtype A (IA; T1a, N0, M0) of primary cancer but without manifesting distant metastasis were enrolled into the multicentered, randomized, double‐blinded, and controlled pilot studies. An age range of 40–75 years with histologically proven PCa was required for recruitment into the individual clinical cohorts. Data regarding tumor size, histologic type, tumor penetration, lymph node metastasis, and TNM (tumor, node and metastasis) stage were obtained from pathologic records. Before chemotherapy, tumors were acquired from these patients as ‘pre’ samples (‘Untreated’ cohort). After chemotherapy, remaining tumors in patients were acquired as ‘post’ samples (‘chemo‐treated’ cohort; most tumors were collected within 1–6 months after treatment). For some cases, the pre‐ and post‐tumor biopsies from the same individual were both accessible, and these samples were subjected to further evaluation. Tumors were processed as formalin‐fixed and paraffin‐embedded biospecimens and sectioned for histological assessment, with alternatively prepared chunks embedded in optimum cutting temperature compound, frozen, and processed via LCM for gene expression analysis. Specifically, stromal compartments associated with glands and adjacent to cancer epithelium were separately isolated from tumor biopsies before and after chemotherapy using an Arcturus (Veritas Microdissection) laser capture microscope following previously defined criteria (Sun et al. 2012). The immunoreactive scoring (IRS) gives a range of 1–4 qualitative scores according to staining intensity for each tissue sample. Categories for the IRS include: 0–1 (negative), 1–2 (weak), 2–3 (moderate), and 3–4 (strong) (Fedchenko and Reifenrath 2014). The diagnosis of PCa was confirmed based on histological evaluation by independent pathologists. Randomized control trial protocols and all experimental procedures were approved by the Ethics Committee and Institutional Review Board of Shanghai Jiao Tong University School of Medicine, Shanghai Institute of Nutrition and Health, Chinese Academy of Sciences, or Zhongshan Hospital of Fudan University, with methods carried out in accordance with the official guidelines. Informed consent was obtained from all subjects, and the experiments conformed to the principles set out in the World Medical Association Declaration of Helsinki and the Department of Health and Human Services Belmont Report.

### 4.3 Omni‐ATAC Protocol

Cells for each of the CTRL, BLEO, or BLEO/ML324 group (three replicates per group) were pretreated with 200 U mL^−1^ DNase (Worthington) for 30 min at 37°C to remove free‐floating DNA and to digest DNA of dead cells. The media were then washed out; cells were resuspended in cold PBS for counting and processed as previously described (Corces et al. 2017). Briefly, a total of 50,000 cells were resuspended in 1 mL of cold ATAC‐seq resuspension buffer (RSB; 10 mM Tris–HCl (pH 7.4), 10 mM NaCl and 3 mM MgCl_2_ in water). Cells were centrifuged at 500 r.c.f. for 5 min in a pre‐chilled (4°C) fixed‐angle centrifuge. After centrifugation, 900 μL of supernatant was aspirated, and the remaining 100 μL of supernatant was carefully aspirated by pipetting with a P200 pipette tip to avoid the cell pellet. Cell pellets were then resuspended in 50 μL of ATAC‐seq RSB containing 0.1% NP40, 0.1% Tween‐20, and 0.01% digitonin by pipetting up and down thrice. This cell lysis reaction was incubated on ice for 3 min. After lysis, 1 mL of ATAC‐seq RSB containing 0.1% Tween‐20 (without NP40 or digitonin) was added, and the tubes were inverted to mix. Nuclei were then centrifuged for 10 min at 500 r.c.f. in a pre‐chilled (4°C) fixed‐angle centrifuge. Supernatant was removed with two pipetting steps, as described before, and nuclei were resuspended in 50 μL of transposition mix (25 μL 2 × TD buffer, 2.5 μL transposase (100 nM final), 16.5 μL PBS, 0.5 μL 1% digitonin, 0.5 μL 10% Tween‐20, and 5 μL H_2_O) by pipetting up and down six times. The remainder of the ATAC‐seq library preparation was performed by following the manufacturer's instructions (Nextera DNA Flex Library Prep kit; Illumina, 20,018,704), and the sample quality was validated using the BioAnalyzer 2100 (Agilent) before proceeding to deep sequencing. Briefly, all libraries were amplified with a target concentration of 20 μL at 4 nM, which is equivalent to 80 fmole of product. Libraries were purified with the 1.5 × AMPure (Beckman) beads and were subjected to next‐generation sequencing. Raw data were deposited in the NCBI Gene Expression Omnibus (GEO) database under the accession code GSE135481.

### 4.4 ATAC‐Seq Data Processing

ATAC‐seq data analyses were performed by DIATRE Biotechnology. The single‐end ATAC‐seq reads were aligned to the hg38 reference genome with random chromosomes cleaned using Bowtie (version 2.2.2) (Langmead and Salzberg 2012) under the parameters ‐t ‐q ‐N 1 ‐L 25. The paired‐end ATAC‐seq reads were aligned with the parameters: ‐t ‐q ‐N 1 ‐L 25 ‐X 2000 ‐no‐mixed–no‐discordant. All unmapped reads, non‐uniquely mapped reads, and PCR duplicates were removed. For downstream analysis, we normalized the read counts by computing the numbers of RPKM values. RPKM values were averaged for each bin between replicates. To minimize the batch and cell type variation, the RPKM values were further normalized by *z*‐score transformation. To visualize the ATAC‐seq signal in the UCSC genome browser, we extended each read by 250 base pairs (bp) and counted the coverage for each base. The correlation between ATAC‐seq replicates was calculated as follows: each read was extended 250 bp from the mapped end position, and the RPKM value was generated on a 100‐bp window base. The ATAC‐seq enrichment was then summed within each 2‐kb window for the entire genome and was compared between replicates. Pearson's correlation was calculated. In brief, to assign each read to its parental origins, we examined all single‐nucleotide polymorphisms (SNPs) in the read that showed high‐quality base calling (Phred score ≥ 30). For paired‐end reads, SNP information from both reads in the pair was summed and used. When multiple SNPs were present in a read (or a read pair), the parental origin was determined by votes from all SNPs, and the read was assigned to the allele that had at least two thirds of the total votes.

### 4.5 Study Approval

All animal experiments were conducted in compliance with the National Institutes of Health (NIH) Guide for the Care and Use of Laboratory Animals and the ARRIVE guidelines, and were approved by the Institutional Animal Care and Use Committee (IACUC) of Shanghai Institute of Nutrition and Health, Chinese Academy of Sciences (no. SINH‐2020‐SY‐1). Mice were maintained under specific pathogen‐free (SPF) conditions, with food and water provided ad libitum.

### 4.6 Statistics

Unless otherwise specified, data in the figures are presented as the mean ± SD, and statistical significance was determined using an unpaired two‐tailed Student's *t*‐test (for comparing two groups), one‐way ANOVA or two‐way ANOVA (for comparing more than two groups), Pearson's correlation coefficients test, Kruskal–Wallis, log‐rank test, Wilcoxon‐Mann–Whitney *U*‐test or Fisher's exact test with GraphPad Prism 8.0 primed with customized parameters. Cox proportional hazards regression model and multivariate Cox proportional hazards model analysis were performed with statistical software SPSS. To determine sample size, we chose to set the values of type I error (*α*) and power (1‐*β*) to be statistically adequate: 0.05 and 0.80, respectively (Krzywinski and Altman 2013). We then determined *n* on the basis of the smallest effect we wish to measure. If the required sample size was too large, we chose to reassess the objectives or to more tightly control the experimental conditions to reduce the variance. For all statistical tests, a *p* value < 0.05 was considered significant, with *p* values presented as digital numbers. The usage of all statistical approaches was examined by biostatistics experts. All bioinformatics analysis and comparisons are described in sufficient detail.

## Author Contributions

Y.S. and B.Z. conceived this study, designed the experiments, and supervised the project. B.Z. performed most of the biological experiments. Q.L. acquired and analyzed clinical samples from patients with PCa and managed participant information. L.H. and Y.S. performed data mining and bioinformatics of gene expression and signaling pathways. S.W., Q.X., S.S., L.H., M.Q., X.R., H.L., and J.J. helped in vitro culture and phenotypic characterization of cancer cells. J.G., X.Z., X.C., Q.F., E.W.‐F.L., J.C., and J.L.K. provided conceptual inputs or supervised a specific subset of experiments. B.Z., Q.F., and Y.S. performed preclinical studies. Y.S. orchestrated data integration and prepared the manuscript. All authors critically read and commented on the final manuscript.

## Conflicts of Interest

The authors declare no conflicts of interest.

## Supporting information


**Appendix S1:** acel70194‐sup‐0001‐AppendixS1.pdf.

## Data Availability

Requests for further information and reagents should be directed to and will be fulfilled by the corresponding author. Databases used in this study, including TCGA (https://portal.gdc.cancer.gov/) and KEGG (https://www.genome.jp/kegg/) are accessible through publicly released links. RNA‐seq data have been deposited in the GEO under accession numbers GSE128282 and GSE160091. ATAC‐seq data are deposited in the GEO under accession number GSE135481. ChIP‐seq data are deposited in the GEO under accession number GSE163105.
