# Supplementary material for: KDM4 Orchestrates Epigenomic Remodeling of Senescent Cells and Potentiates the Senescence‐Associated Secretory Phenotype
Source: Aging Cell. 2025 Aug 24;24(10):e70194. doi: 10.1111/acel.70194 (PMC12507408; doi:10.1111/acel.70194)
Supplement: Supplementary file 1 — Appendix S1: acel70194‐sup‐0001‐AppendixS1.pdf. [file ACEL-24-e70194-s001.ppt]

## Slide 1
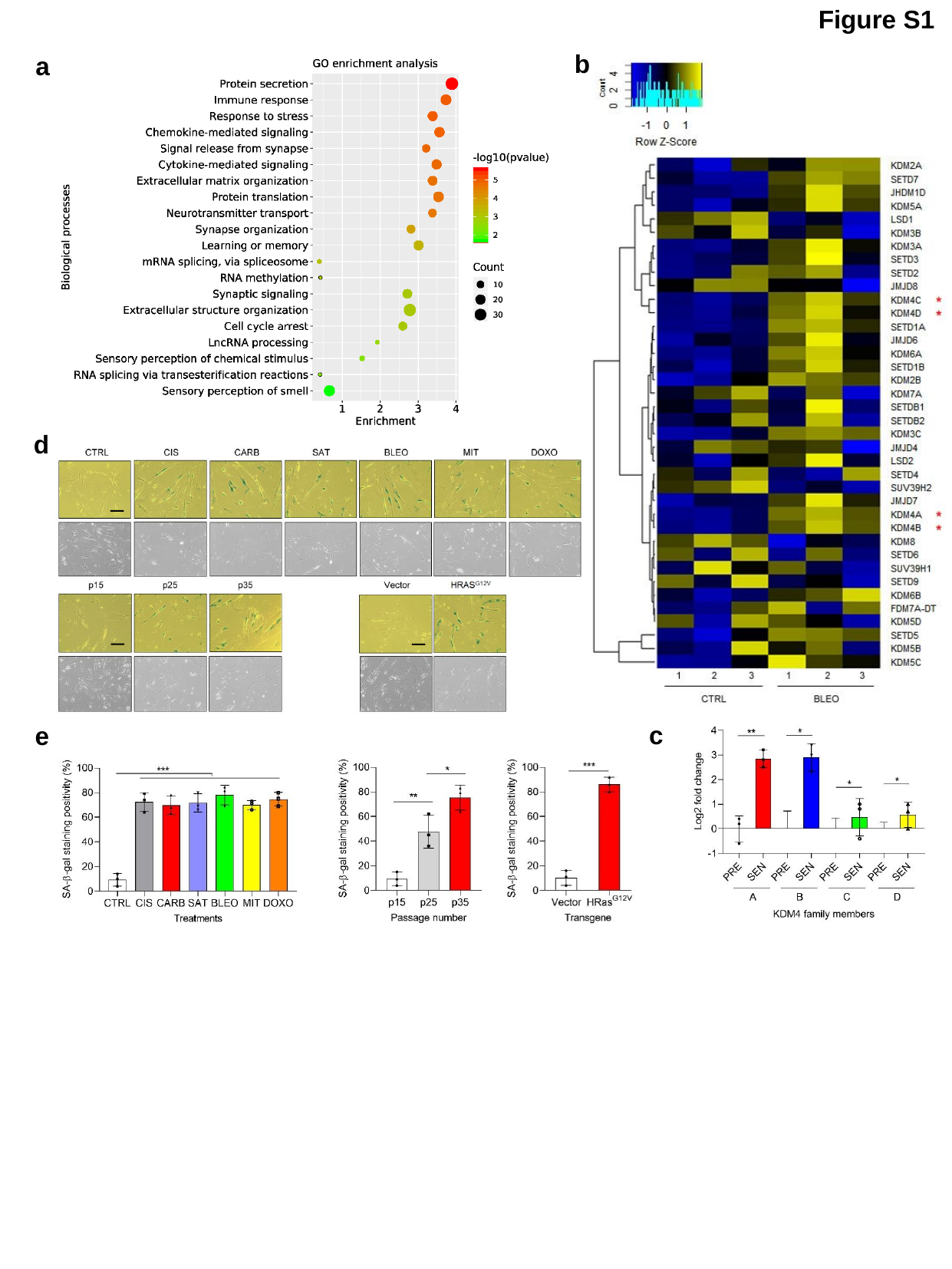

Figure S1
b
a
d
c
e

## Slide 2
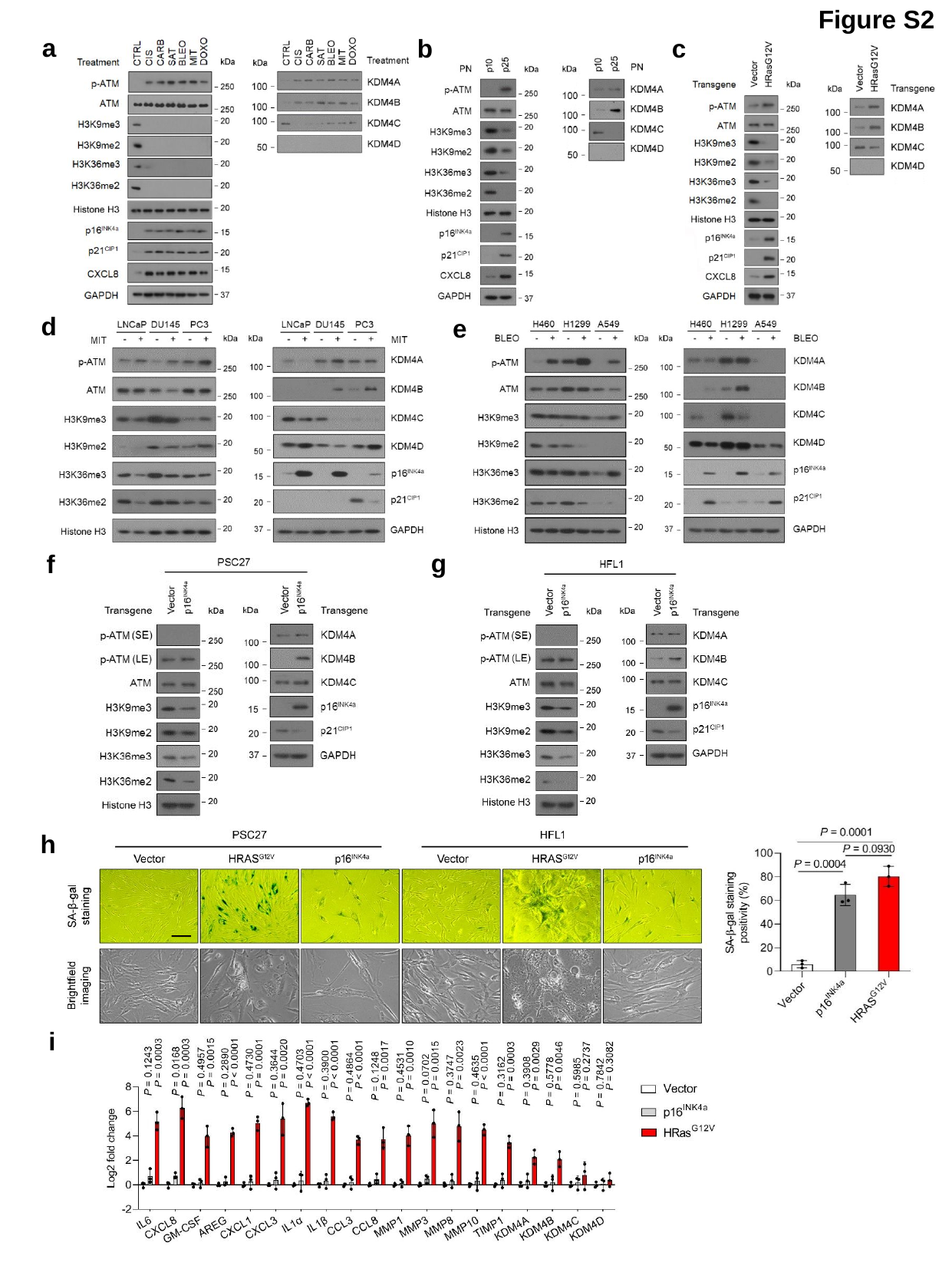

Figure S2
a
c
b
d
e
g
f
h
i

## Slide 3
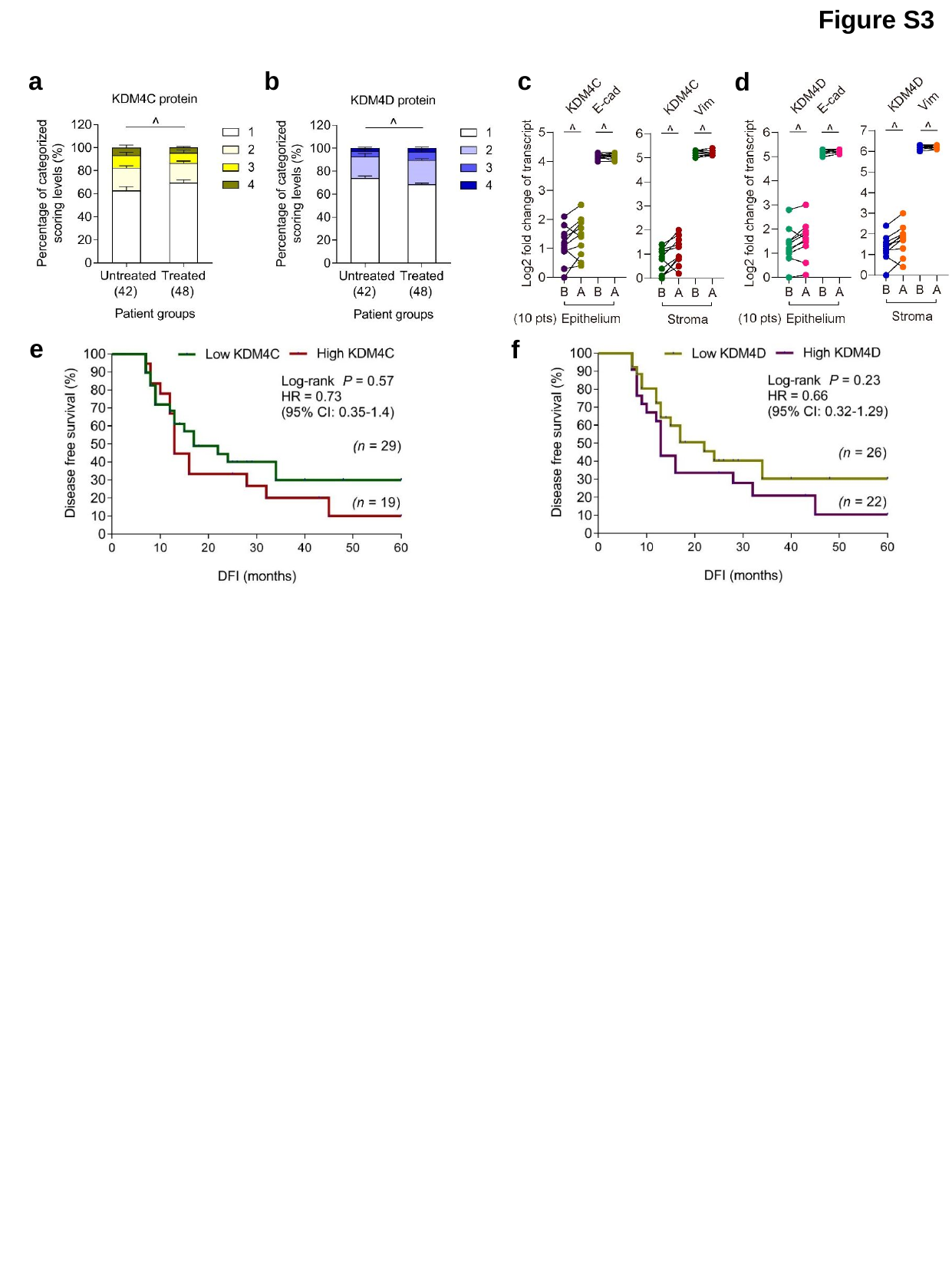

Figure S3
b
a
c
d
e
f

## Slide 4
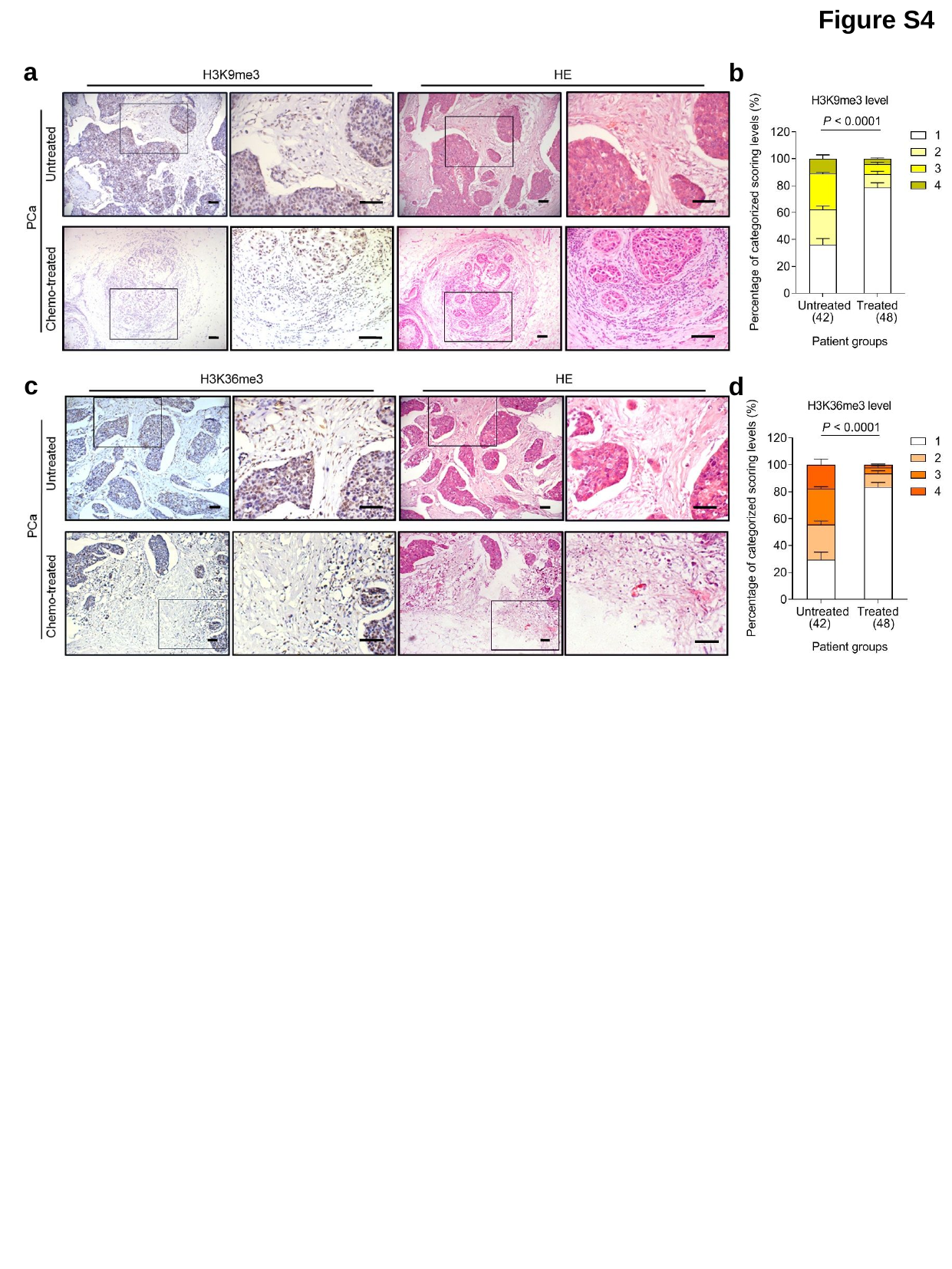

Figure S4
a
b
c
d

## Slide 5
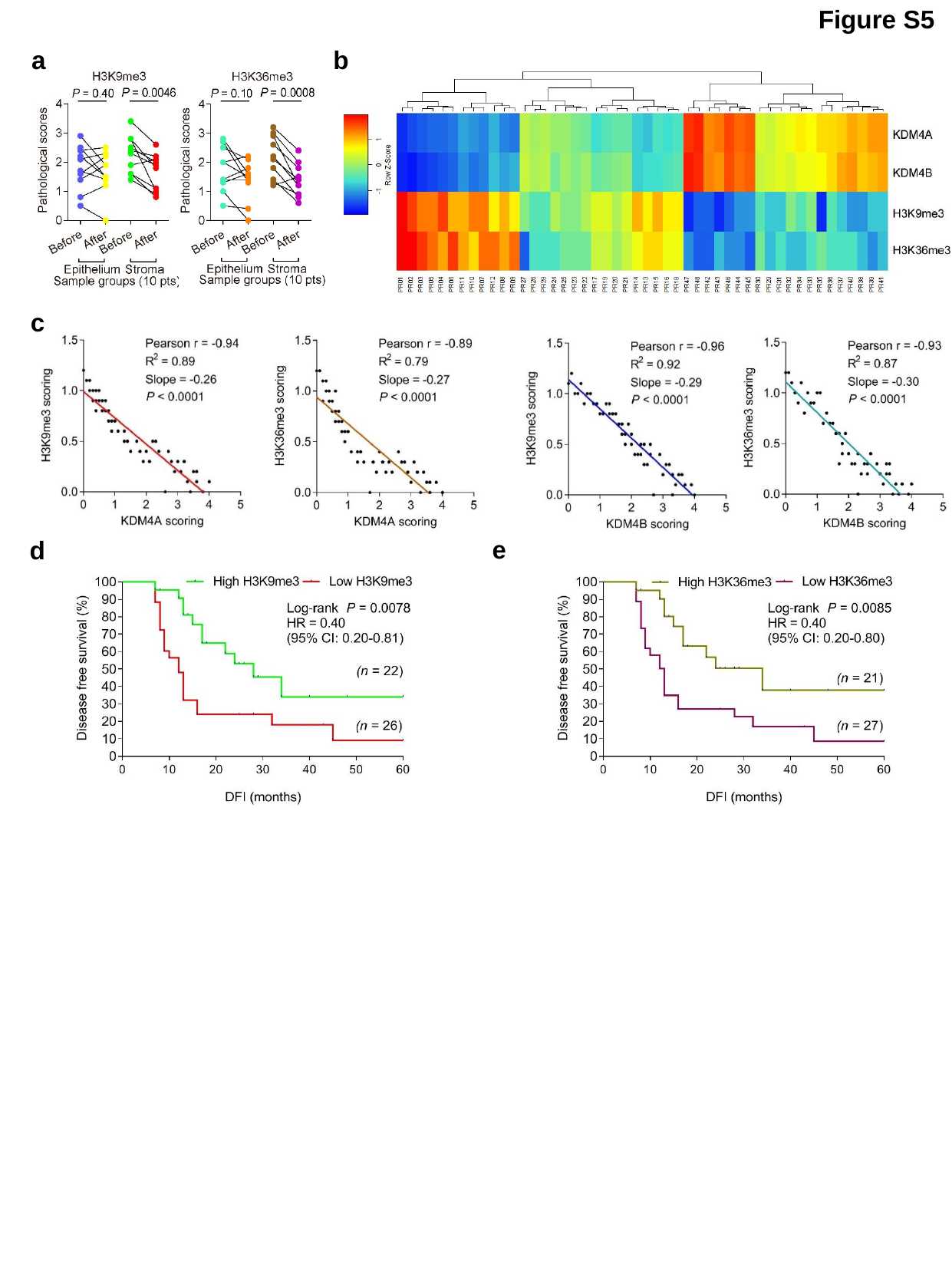

Figure S5
b
a
c
e
d

## Slide 6
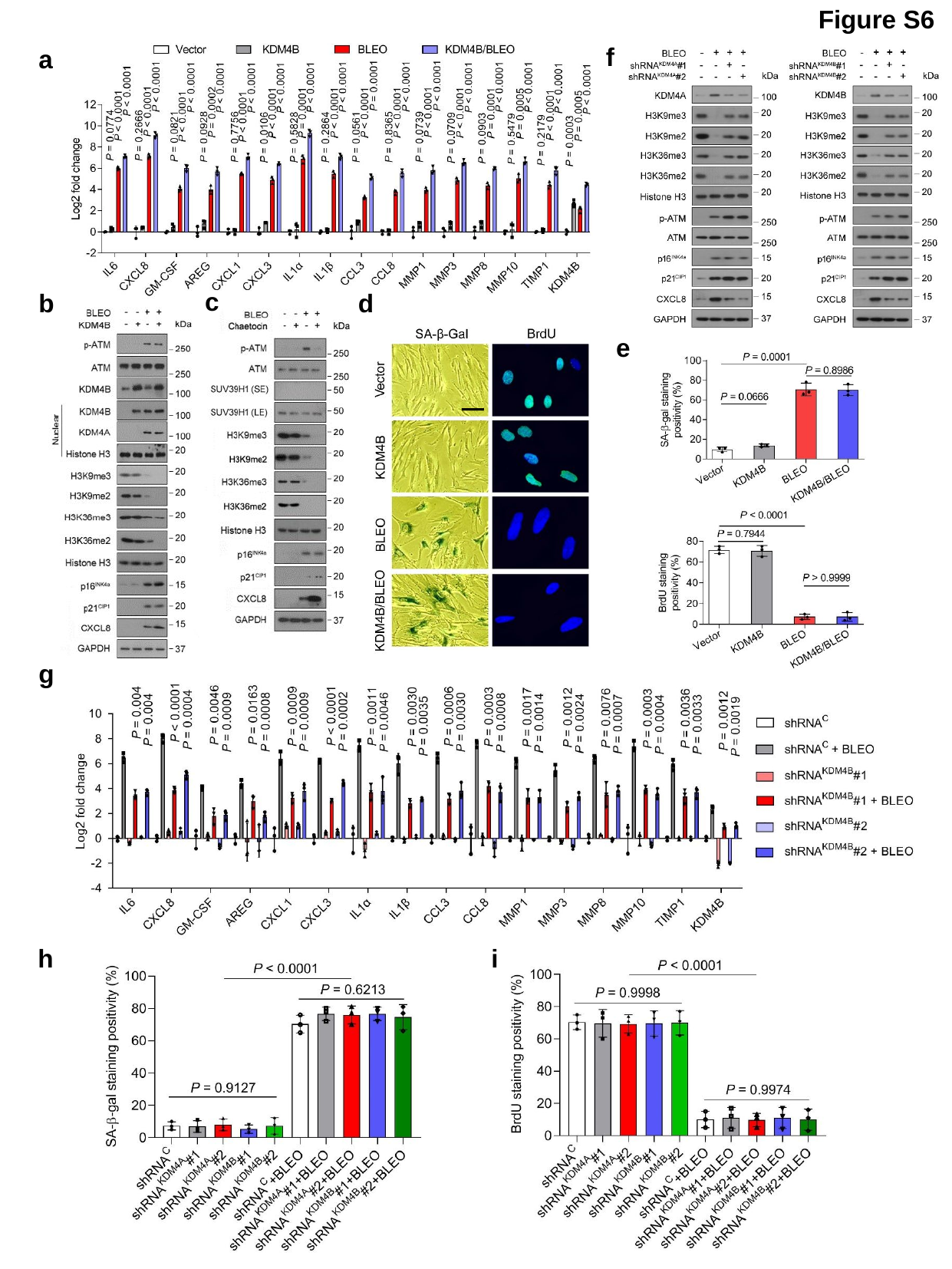

Figure S6
f
a
c
d
b
e
g
h
i

## Slide 7
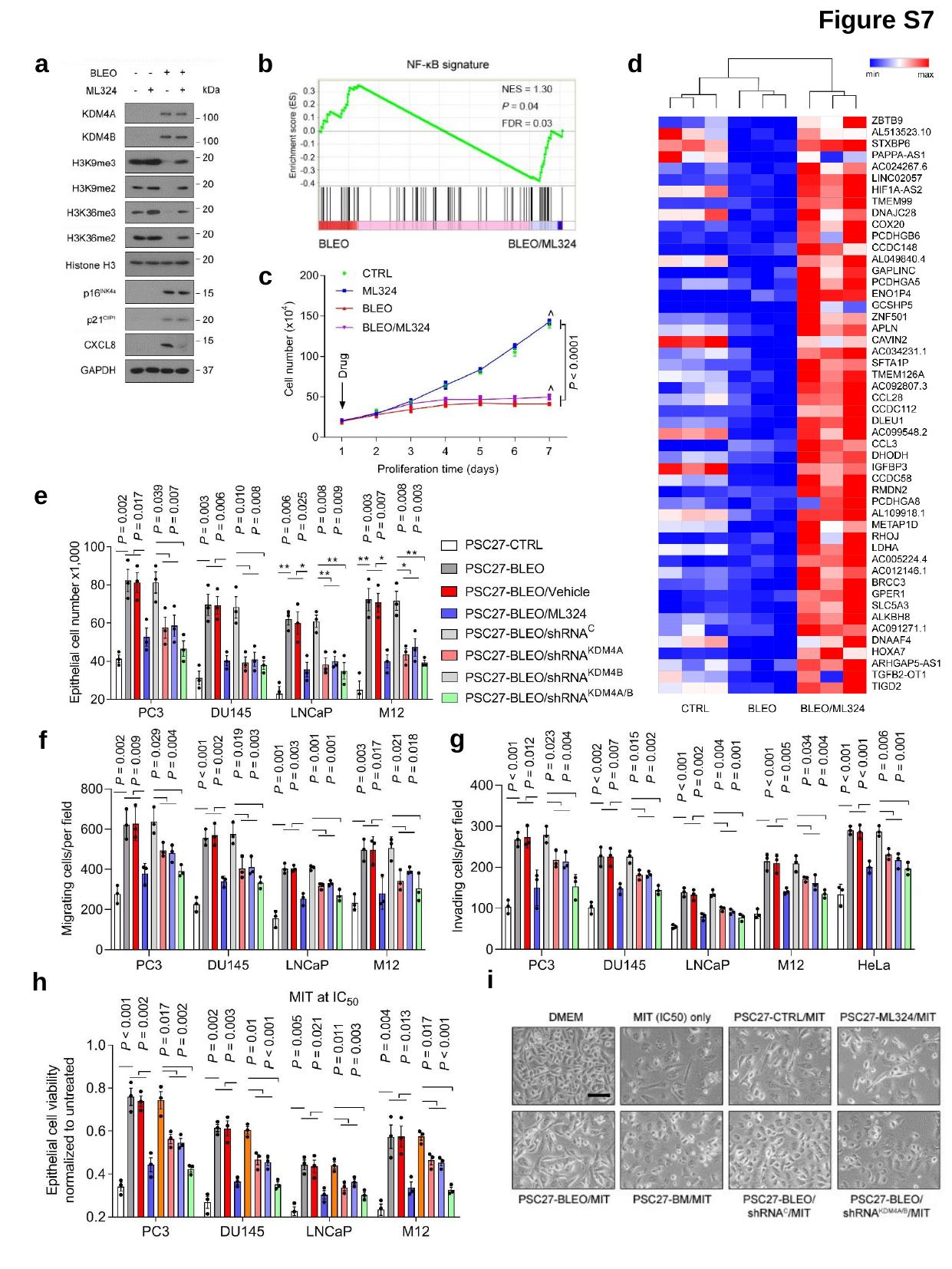

Figure S7
a
b
d
c
e
g
f
i
h

## Slide 8
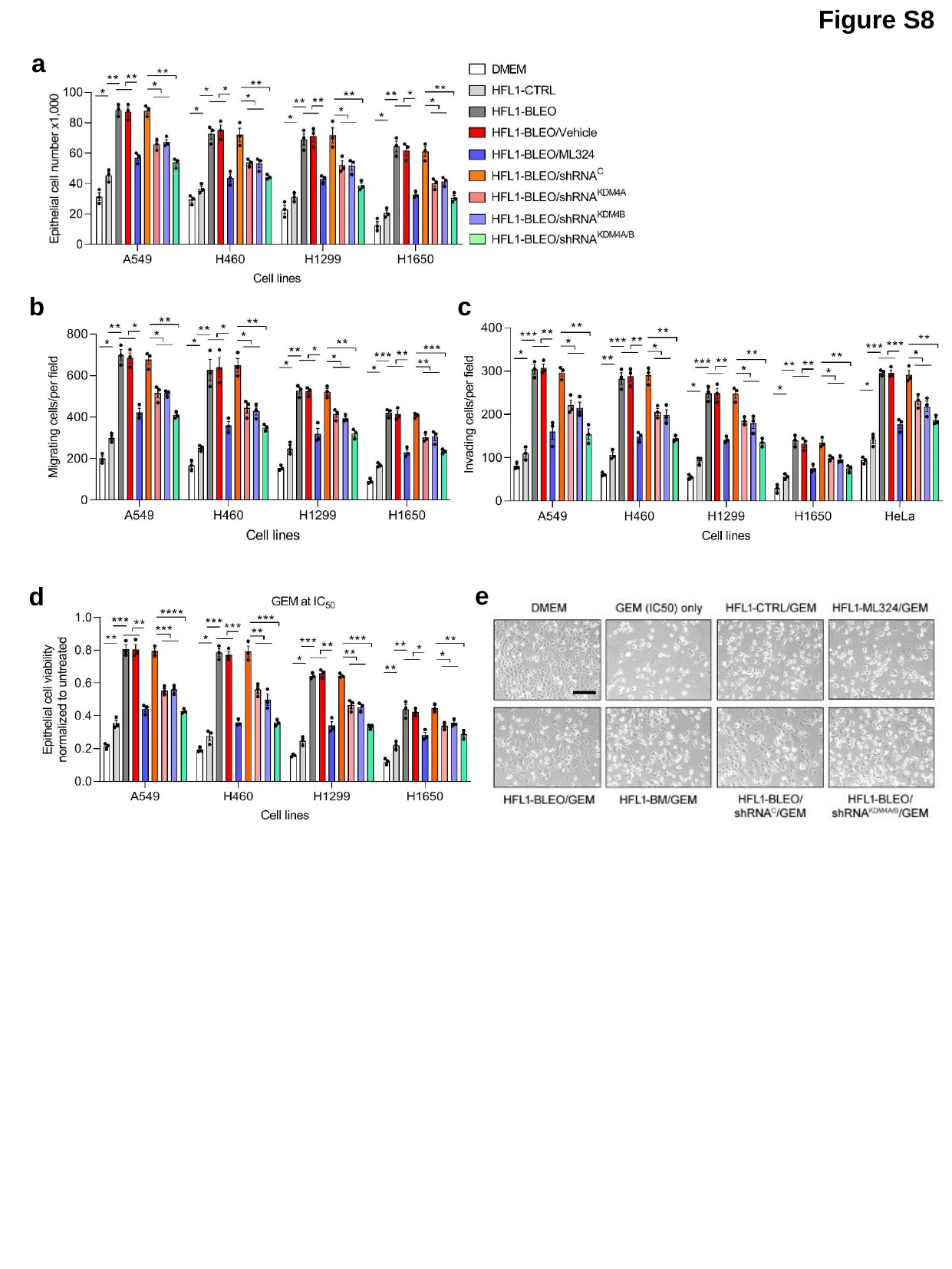

Figure S8
a
c
b
e
d

## Slide 9
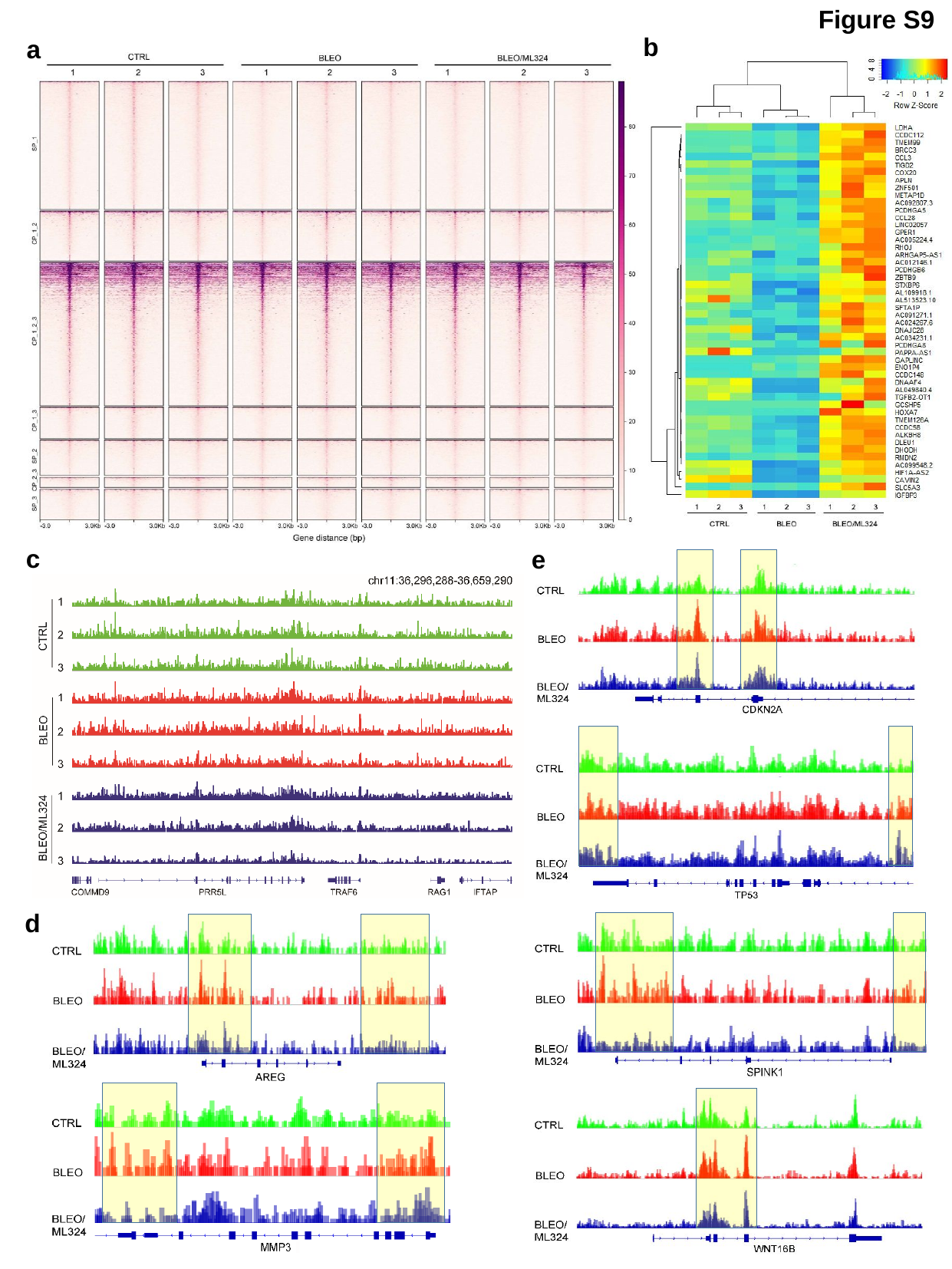

Figure S9
b
a
c
e
d

## Slide 10
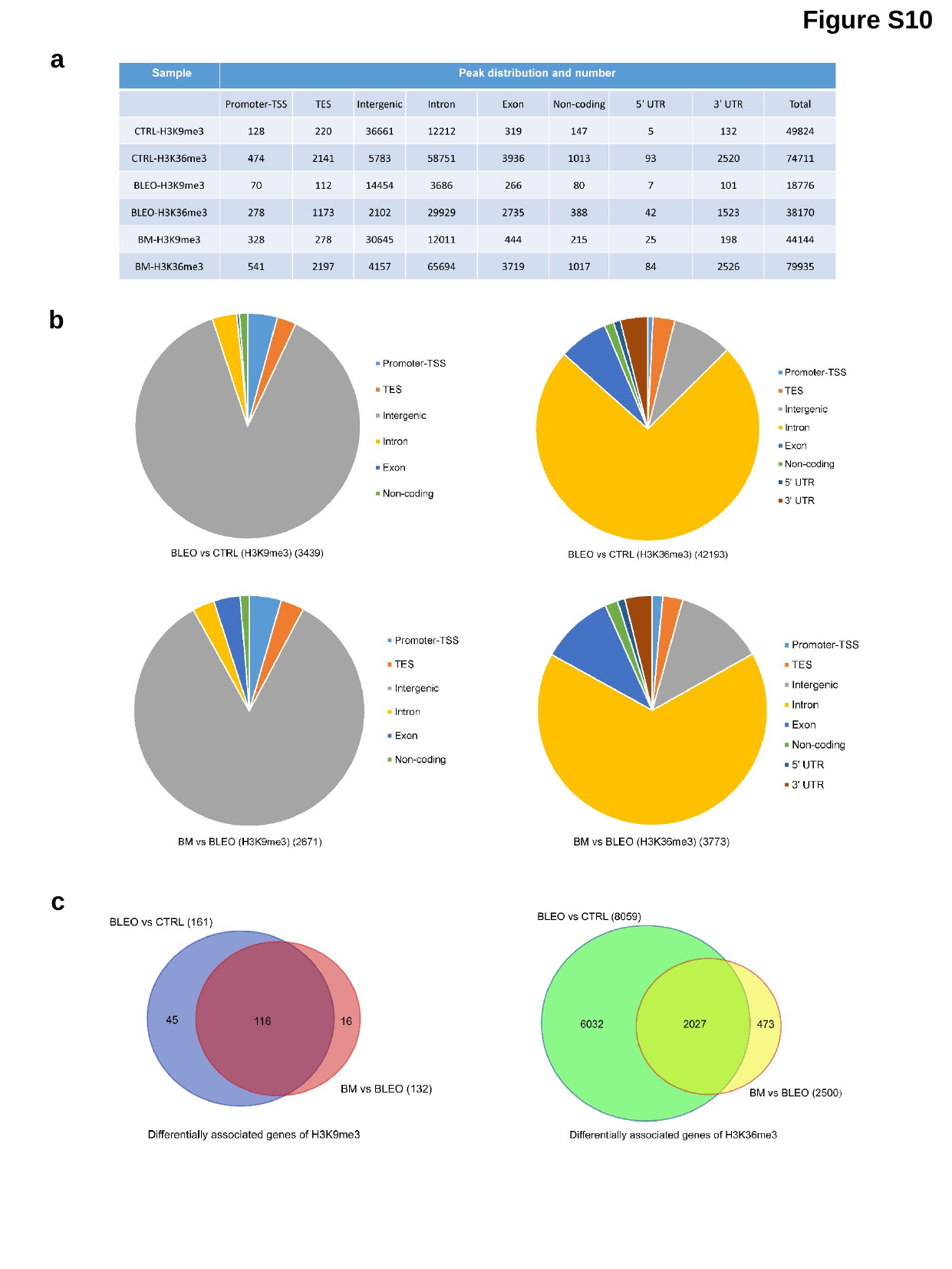

Figure S10
a
b
c

## Slide 11
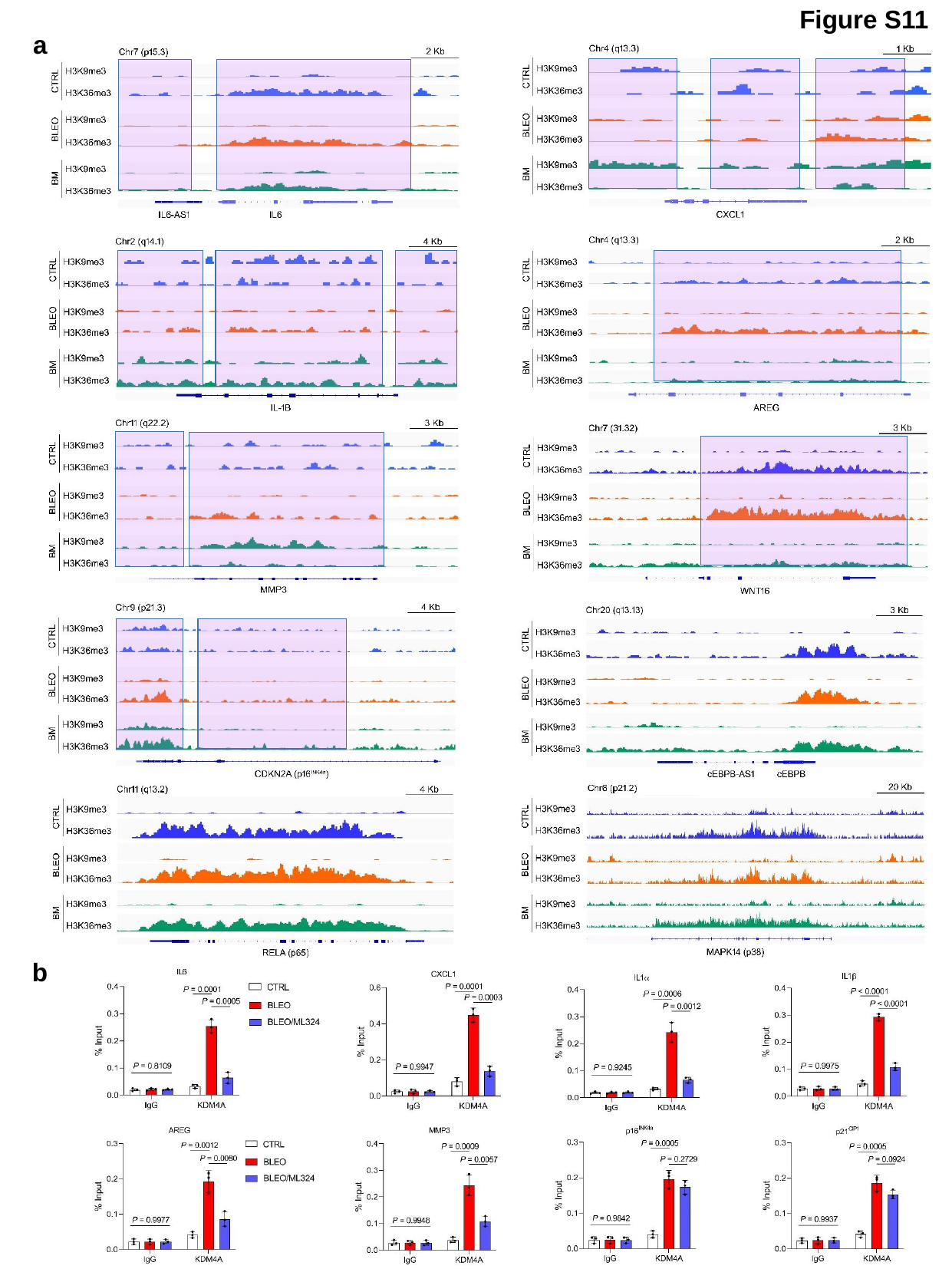

Figure S11
a
b

## Slide 12
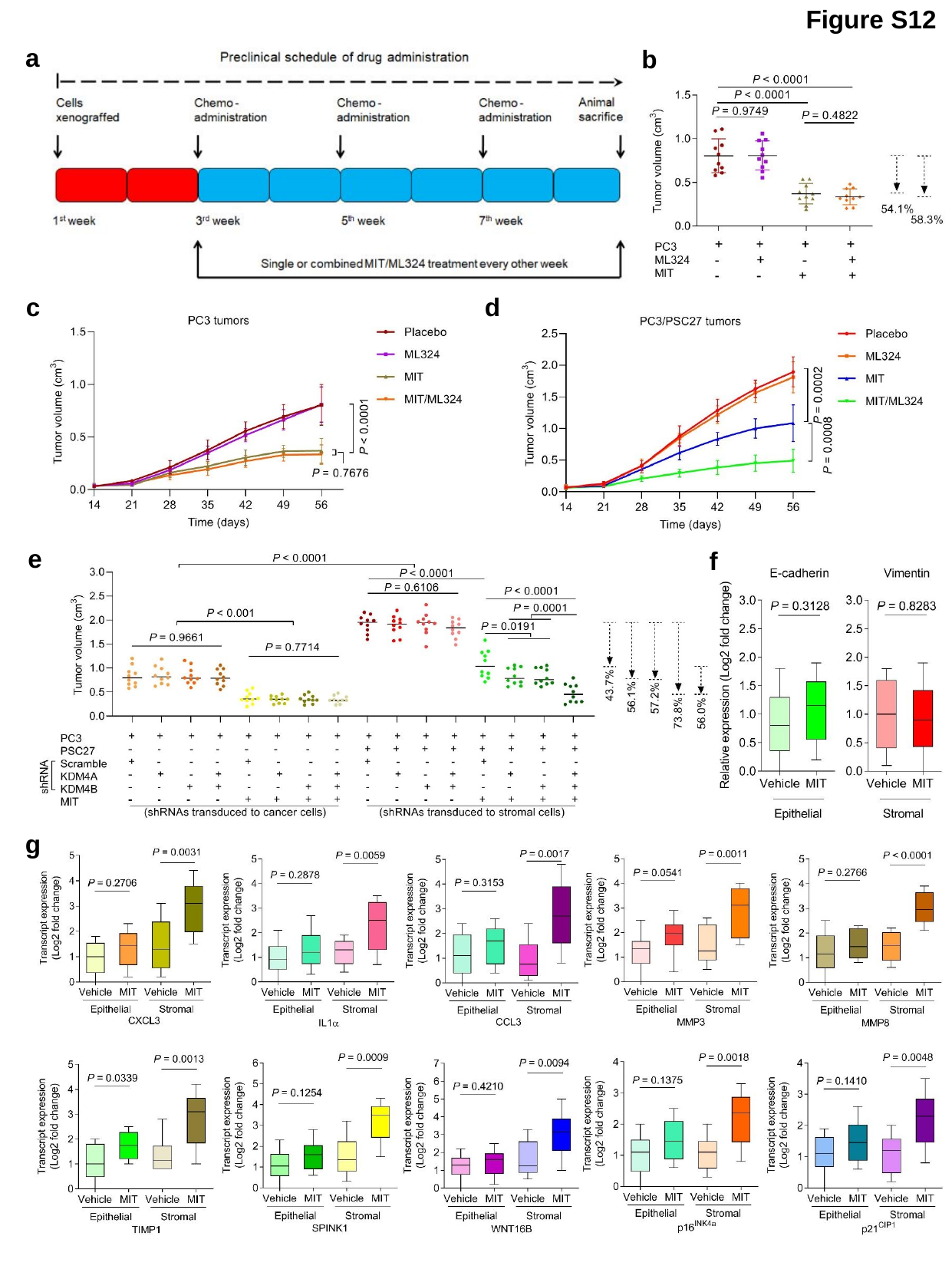

Figure S12
a
b
d
c
e
f
g

## Slide 13
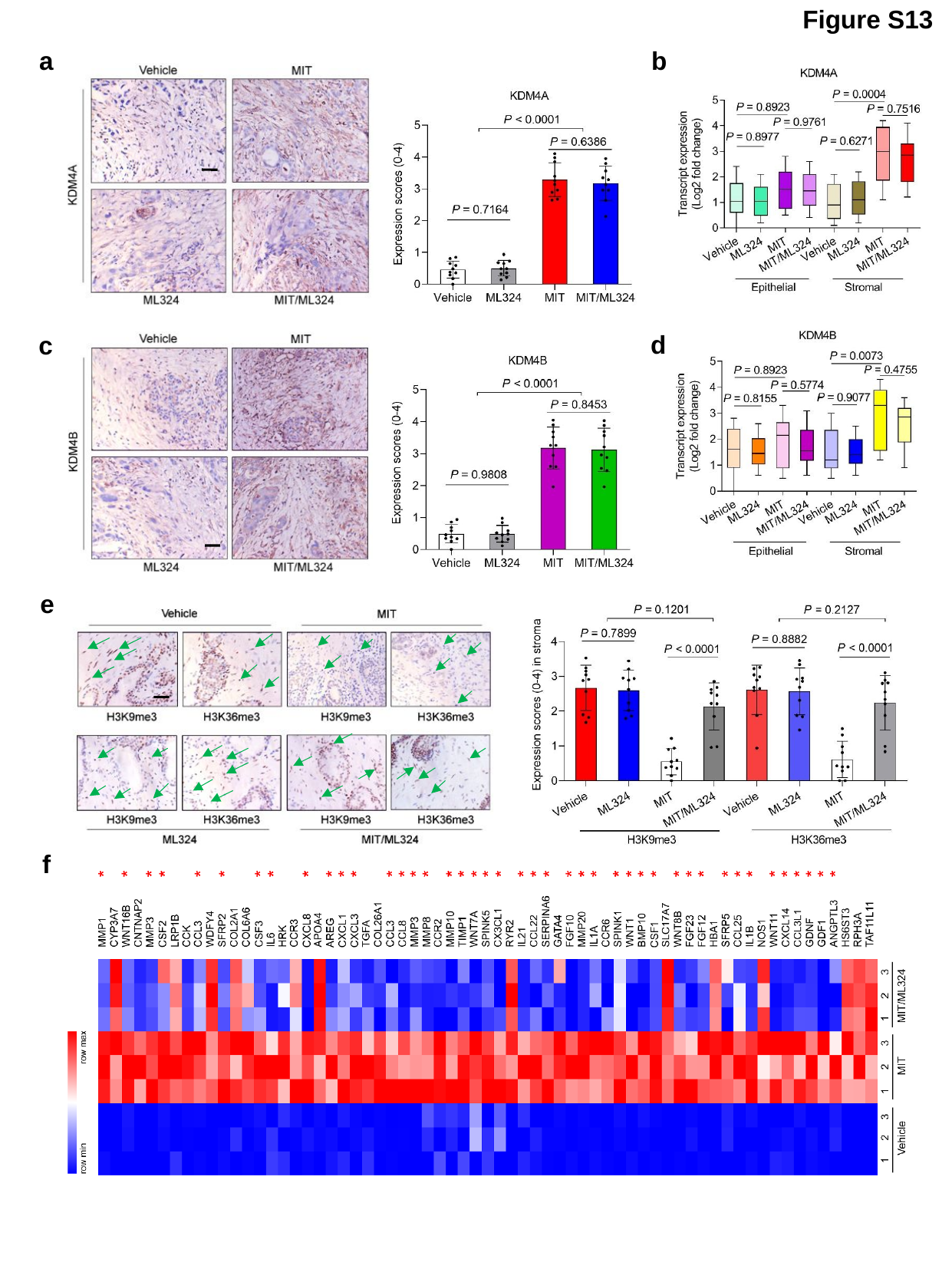

Figure S13
b
a
d
c
e
f

## Slide 14
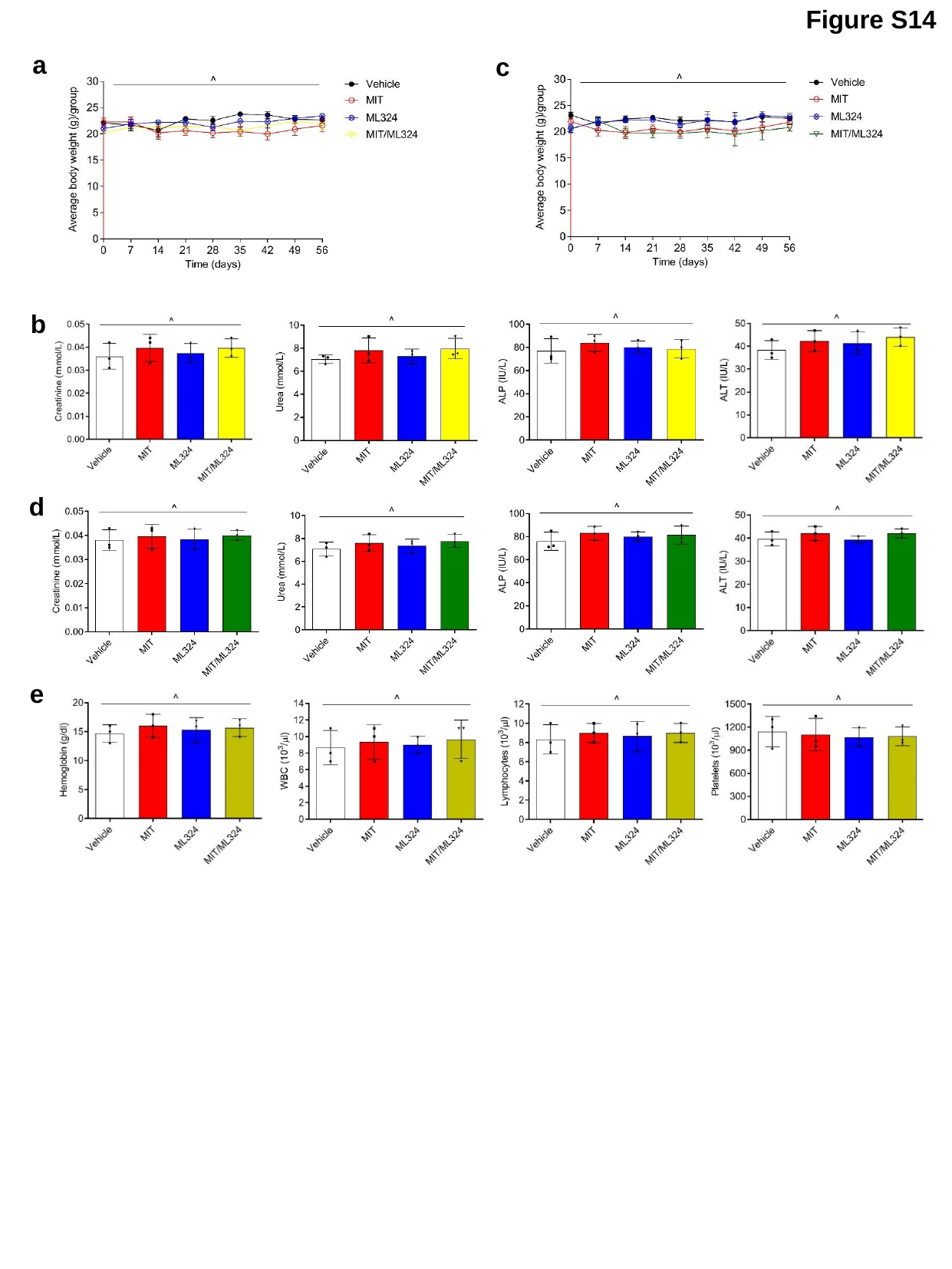

Figure S14
a
c
b
d
e

## Slide 15
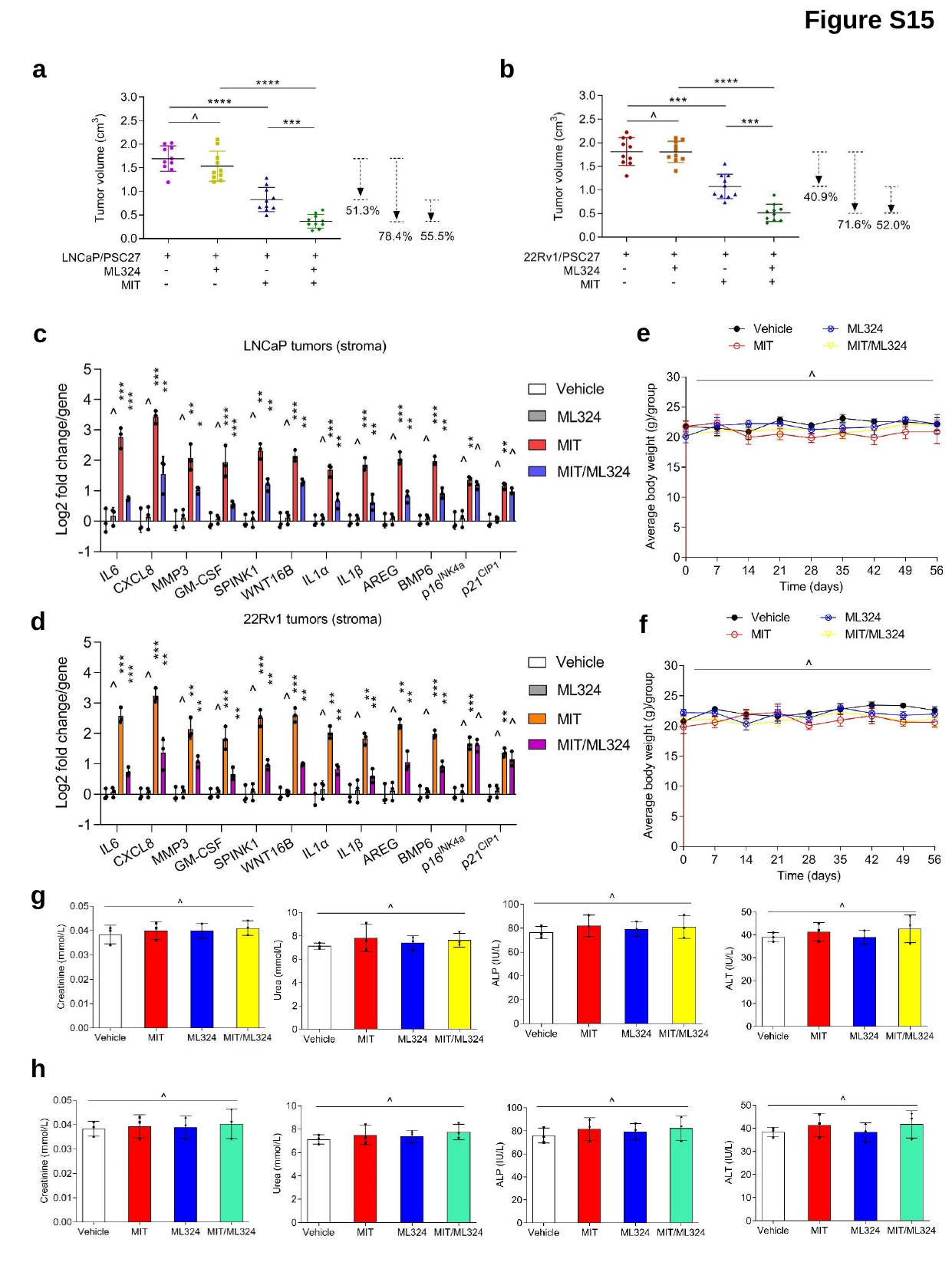

Figure S15
b
a
e
c
d
f
g
h

## Slide 16
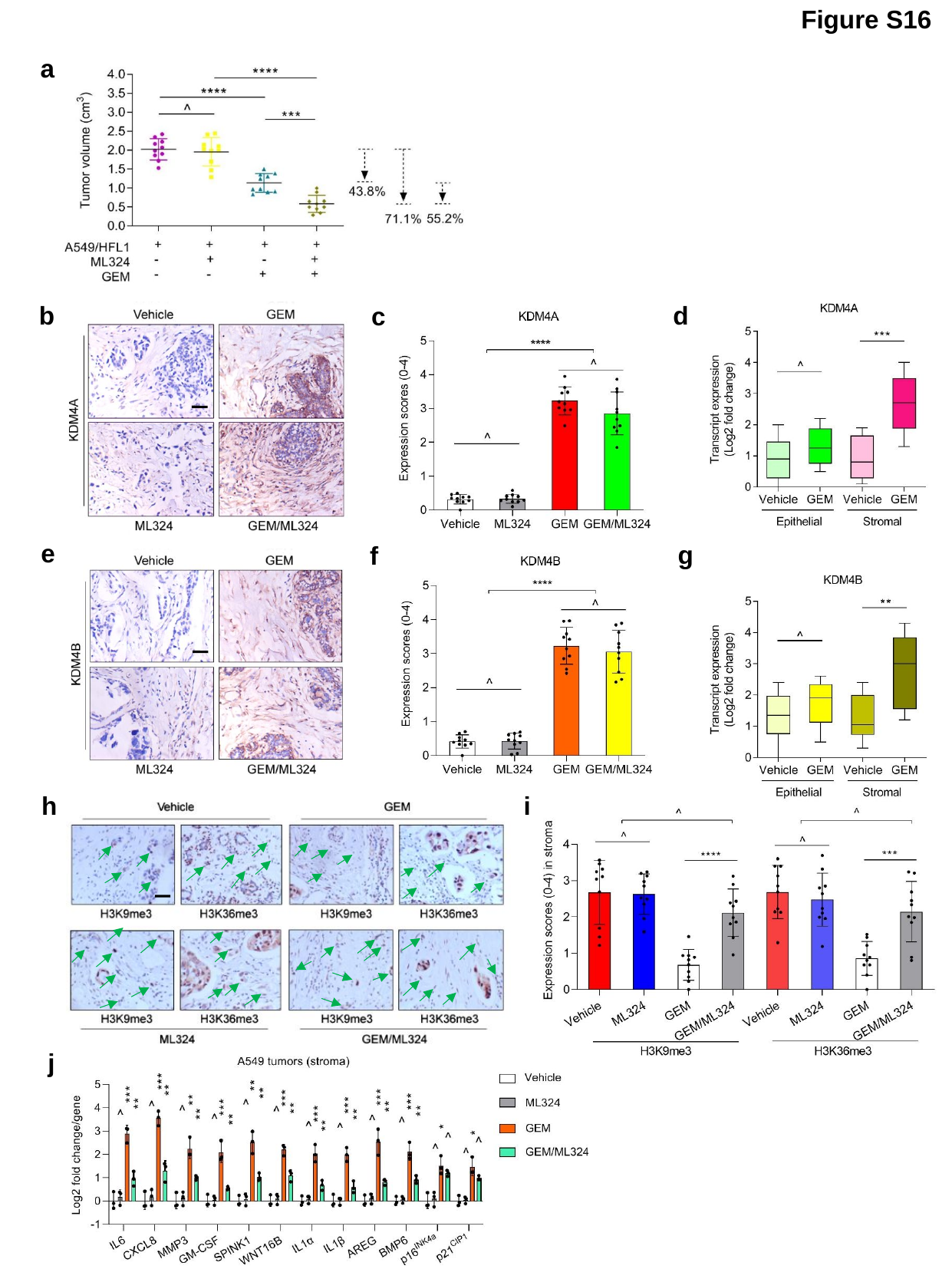

Figure S16
a
b
d
c
e
g
f
h
i
j

## Slide 17
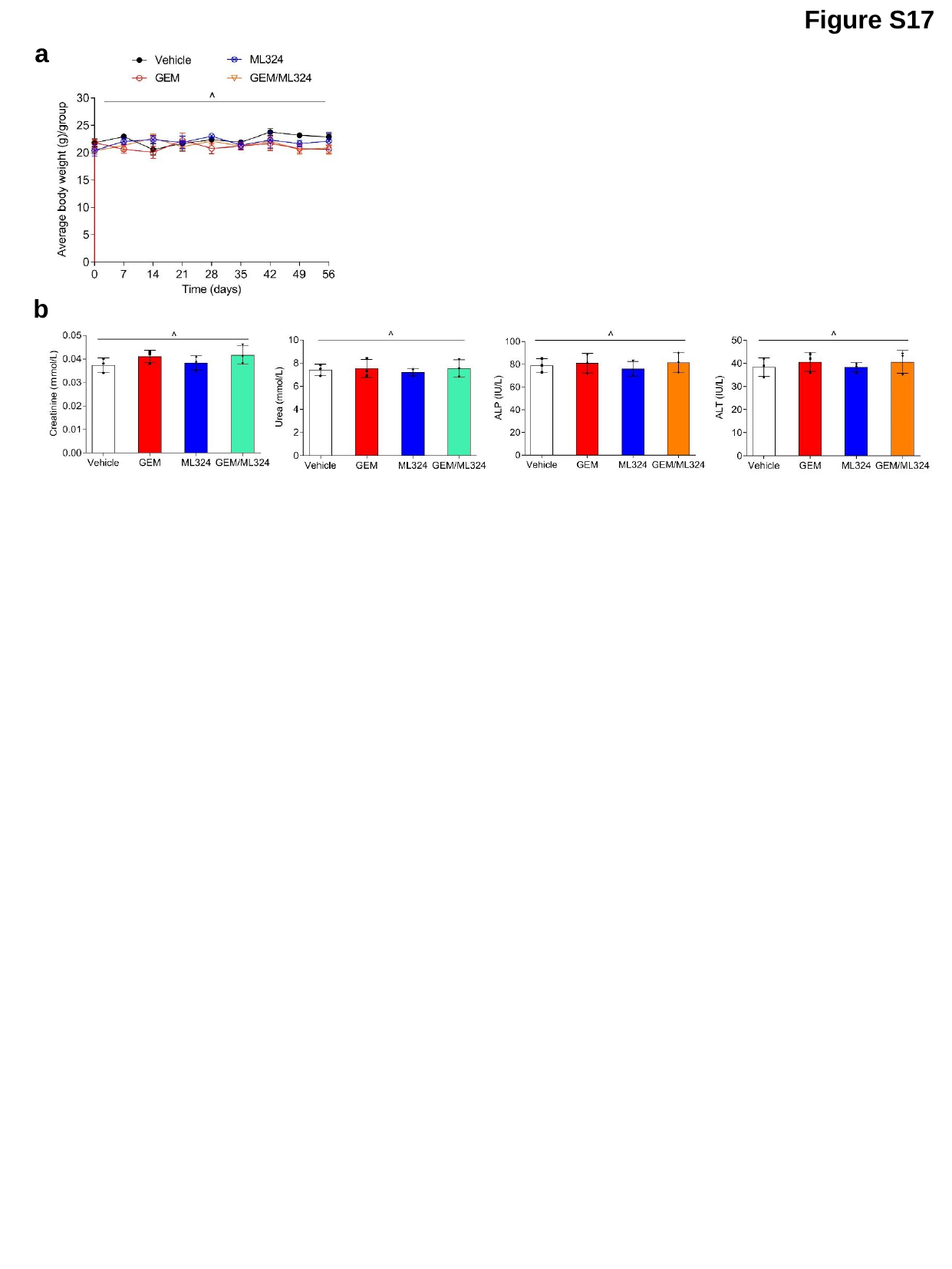

Figure S17
a
b

## Slide 18
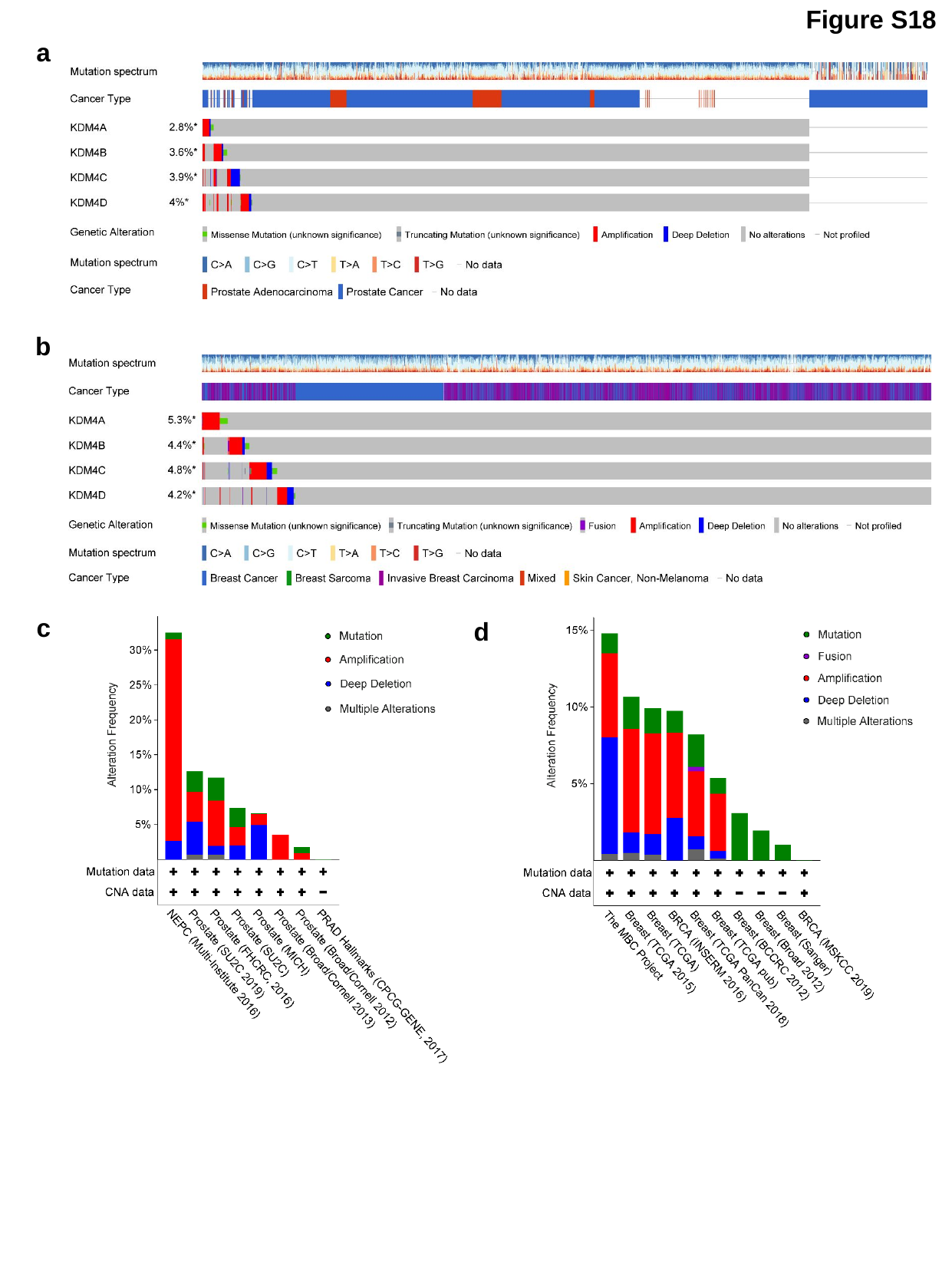

Figure S18
a
b
c
d
